# Preventive methylene blue treatment preserves cognition in mice expressing full-length pro-aggregant human Tau

**DOI:** 10.1186/s40478-015-0204-4

**Published:** 2015-05-10

**Authors:** Katja Hochgräfe, Astrid Sydow, Dorthe Matenia, Daniela Cadinu, Stefanie Könen, Olga Petrova, Marcus Pickhardt, Petra Goll, Fabio Morellini, Eckhard Mandelkow, Eva-Maria Mandelkow

**Affiliations:** DZNE (German Center for Neurodegenerative Diseases), Ludwig-Erhard-Allee 2, 53175 Bonn, Germany; CAESAR Research Center, Ludwig-Erhard-Allee 2, 53175 Bonn, Germany; MPI for Metabolism Research, Hamburg Outstation, c/o DESY, Notkestr. 85, 22607 Hamburg, Germany; Center for Molecular Neurobiology Hamburg (ZMNH), Falkenried 94, 20251 Hamburg, Germany

**Keywords:** Alzheimer disease, Tau mouse model, Behavior, Methylene blue, Therapy

## Abstract

**Introduction:**

Neurofibrillary tangles (NFT) composed of Tau are hallmarks of neurodegeneration in Alzheimer disease. Transgenic mice expressing full-length pro-aggregant human Tau (2N4R Tau-ΔK280, termed Tau^ΔK^) or its repeat domain (TauRD-ΔK280, TauRD^ΔK^) develop a progressive Tau pathology with missorting, phosphorylation, aggregation of Tau, loss of synapses and functional deficits. Whereas TauRD^ΔK^ assembles into NFT concomitant with neuronal death, Tau^ΔK^ accumulates into Tau pretangles without overt neuronal loss. Both forms cause a comparable cognitive decline (with onset at 10mo and 12mo, respectively), which is rescued upon switch-off of transgene expression. Since methylene blue (MB) is able to inhibit Tau aggregation in vitro, we investigated whether MB can prevent or rescue Tau-induced cognitive impairments in our mouse models. Both types of mice received MB orally using different preventive and therapeutic treatment protocols, initiated either before or after disease onset. The cognitive status of the mice was assessed by behavior tasks (open field, Morris water maze) to determine the most successful conditions for therapeutic intervention.

**Results:**

Preventive and therapeutic MB application failed to avert or recover learning and memory deficits of TauRD^ΔK^ mice. Similarly, therapeutic MB treatment initiated after onset of cognitive impairments was ineffective in Tau^ΔK^ mice. In contrast, preventive MB application starting before onset of functional deficits preserved cognition of Tau^ΔK^ mice. Beside improved learning and memory, MB-treated Tau^ΔK^ mice showed a strong decrease of insoluble Tau, a reduction of conformationally changed (MC1) and phosphorylated Tau species (AT180, PHF1) as well as an upregulation of protein degradation systems (autophagy and proteasome). This argues for additional pleiotropic effects of MB beyond its properties as Tau aggregation inhibitor.

**Conclusions:**

Our data support the use of Tau aggregation inhibitors as potential drugs for the treatment of AD and other tauopathies and highlights the need for preventive treatment before onset of cognitive impairments.

**Electronic supplementary material:**

The online version of this article (doi:10.1186/s40478-015-0204-4) contains supplementary material, which is available to authorized users.

## Introduction

Alzheimer disease (AD) is a fatal dementia with progressive accumulation of protein aggregates composed of amyloid-beta (Aβ) and the microtubule-associated Tau protein in brain structures relevant for learning and memory [1]. Despite much progress made in recent years, the details of the neurotoxic cascade are still a matter of debate. However, it is assumed that the accumulation of protein aggregates is closely linked to neurotoxicity and degeneration. Current treatment options for AD are still limited to acetylcholinesterase inhibitors (donepezil, galantamine, rivastigmine), NMDA (N-methyl-D-aspartate) receptor antagonists (memantine) or other supportive strategies ameliorating the symptoms, rather than treating the underlying causes of neurodegeneration [2]. Recently, a number of promising drug candidates targeting different stages of Aβ pathology failed in clinical trials [3,4]. In addition the spreading of Tau pathology (rather than that of Aβ pathology) correlates closely with loss of synapses and neurons, the best correlate of cognitive decline [5-7]. As a result, Tau has become a promising target for therapeutic intervention.

The process of Tau aggregation, from oligomers to neurofibrillary tangles (NFT) is suspected to induce neurodegeneration. Support for this hypothesis came from Tau transgenic mouse models expressing aggregation-prone Tau mutants. Examples are P301S [8-10], P301L [11-13] and ΔK280 [14,15], or, as negative controls, non-aggregating Tau mutants, e.g. ΔK280-PP [16,17]. Aggregation-prone Tau mutants were identified from cases of sporadic and hereditary forms of frontotemporal dementia (FTD). The deletion mutation ΔK280 was originally detected in a sporadic FTD case [18,19] and more recently in an Alzheimer patient [20].

In mice, expression of either full-length pro-aggregant human Tau-ΔK280 (Tau^ΔK^) or its repeat domain (TauRD^ΔK^) under control of the CaMKIIα promoter leads to progressive neuropathology. Both models share several common aspects, including the formation of co-aggregates composed of exogenous human and endogenous mouse Tau, loss of synapses accompanied by functional deficits such as cognitive decline and electrophysiological impairments. The most important similarity of both models is that the functional deficits can be recovered after switching-off the expression of pro-aggregant Tau. As a consequence of switch-off, Tau co-aggregates undergo structural reorganization with release and clearance of exogenous Tau, whereas the aggregated endogenous mouse Tau persists for several months. By contrast, the two models clearly differ in the onset and severity of Tau pathology. TauRD^ΔK^ is the more toxic Tau species, presumably because of its greater propensity for aggregation based on β-structure. Thus, expression of TauRD^ΔK^ leads to early development of silver-positive NFT and neuronal loss inside the hippocampus formation (~5mo), which are nearly absent in Tau^ΔK^ mice. In addition cognitive decline and loss of synapses occurs earlier in TauRD^ΔK^ (~10mo) than in Tau^ΔK^ mice (~12mo).

Despite the variations between the TauRD^ΔK^ and Tau^ΔK^ mouse models, the studies summarized above share the essential fact that tauopathies as such are largely reversible, provided that the amyloidogenic Tau is removed [14,15]. Thus strategies and substances counteracting Tau aggregation are promising candidates for the treatment of AD and other tauopathies.

Several screens identified low molecular weight compounds, which act as Tau aggregation inhibitors [21-24]. One compound of particular interest is methylene blue (MB). The inhibitory effect of MB applies not only to Tau aggregation [24,25] but also to other proteins involved in neurodegeneration such as huntingtin [26], TDP-43 and alpha-synuclein [27], Aβ [28] and prion protein [29]. MB is an FDA approved drug and has a long history of medical use, mainly as an antiseptic and anti-malaria compound. Biochemically, MB is a bioavailable member of the phenothiazine family with high water solubility. It is a redox-cycling compound, relatively non-toxic and able to pass the blood-brain barrier [30,31]. The first anti-Tau therapy with MB in humans was reported by Wischik et al. in 2008 [32]. In this phase 2 clinical trial, a daily dose of 3x 60 mg Rember™ (a derivative of the oxidized form of MB) over 1 year appeared to show a slow-down of cognitive decline in mild and moderate AD patients. Since none of these studies have been published in a peer-reviewed journal, a skeptical attitude towards the presented results remains in the field. Meanwhile Rember™ was replaced by its more sophisticated successor LMTX™, which is optimized in terms of enhanced tolerability and higher absorption in the intestine as it contains the reduced “leuco” form of MB [33]. Up to now three phase-3 trials have been announced for LMTX™, one for the treatment of bvFTD or Pick disease and two studies for AD. First results are expected for late 2015 or early 2016 (as stated by TauRx Therapeutics [34]).

In parallel a number of preclinical studies in various model organisms were performed to elucidate MB’s neuroprotective mechanism of action. However, the results published remain controversial. While MB ameliorates the neurotoxic phenotype in a C. elegans model of tauopathy with aggregated Tau in neurons [35], it was reported to be ineffective in zebrafish expressing P301L mutant Tau without aggregated Tau [36]. In mice, MB showed beneficial phenotypic effects in models of Huntington disease [26] and in models of AD [37-39], but failed in models of amyotrophic lateral sclerosis [40,41]. In addition MB’s effect on neuronal pathology is still under debate. In case of Tau pathology there is a debate on whether MB acts on soluble or insoluble Tau species [39,42,43] and whether additional mechanisms such as enhancing general protein clearance via autophagy or proteasomes, improving energy metabolism, or other effects on ATP/GTP binding proteins play a role [38,44-46].

The present study aims to contribute to the discussion how MB can ameliorate Tau-induced pathological changes and (more importantly) cognitive impairment in inducible pro-aggregant mice. In addition our purpose is to refine treatment paradigms with respect to treatment initiation, concentration and duration. To this end pro-aggregant Tau^ΔK^ and TauRD^ΔK^ mice were treated orally with MB using different long- and short-term treatment protocols to determine the most successful conditions for therapeutic intervention. The effect of MB on cognition was evaluated in different behavior tasks (open field and Morris water maze) and the brain pathology of MB-treated and untreated mice were compared to gain insights into MB’s mode of action.

## Materials and methods

### Generation of pro-aggregant Tau transgenic mice

Transgenic mice expressing pro-aggregant human full-length Tau (2N4R, Tau441, with deletion mutant ΔK280, 441-1 = 440 amino acids, here termed Tau^ΔK^) or pro-aggregant Tau repeat domain (construct K18, 4R, residues 244-372, termed TauRD^ΔK^) were generated as described [16,17] (Additional file 1: Figure S1). Briefly, responder mice carrying either the Tau-transgene together with a luciferase reporter were crossbred with the CaMKIIα-tTA transactivator mice [47] to obtain double-transgenic mice with constitutive expression of luciferase and pro-aggregant Tau^ΔK^ or TauRD^ΔK^. All bigenic offspring were heterozygous and had an identical C57BL/6 genetic background. Non-transgenic littermates were used as controls. The transgene expression of bigenic mice started roughly around birth (0mo) concomitant with the onset of CaMKIIα activity. The expression was measured in vivo by bioluminescence imaging of luciferase activity. All animal procedures were approved in accordance with the German Animal Welfare Act.

### *In vivo* bioluminescence imaging of luciferase activity

*In vivo* bioluminescence imaging to quantify luciferase activity and estimate expression strength of Tau transgenes was performed using an Ivis Lumina II system (Caliper Life Science) as described [15]. Briefly, mice received an intraperitoneal injection of 150 mg/kg D-luciferin/PBS (Caliper Life Science) 10 min prior to imaging and were anesthetized using 2% isoflurane (Abbott). A sequence of images was collected using a highly sensitive CCD camera. The bioluminescence emission was analyzed and quantified by the Living Image 4.0 software (Caliper Life Science).

### Oral methylene blue treatment of Tau transgenic mice

Methylene blue (MB, C_16_H_18_CIN_3_S * 3 H_2_O, Sigma) was administered ad libitum via the drinking water supplemented with saccharin (Huxol, 1 tablet per 200 ml). Mice received a daily MB-dose of 40 or 20 mg/kg based on a daily drinking volume of ~5-6 ml and a body weight of 25-35 g. The concentration of the MB drinking solution was 0.25 mM or 0.5 mM, respectively. Tau^ΔK^ mice were treated using a daily dose of 20 mg/kg MB. In all cases Tau expression started at birth (~0mo); one group of Tau^ΔK^ mice received MB for 14.5mo starting at 1.5mo of age. A second group was administered MB for 6mo, starting at 9mo of age and a third group received MB for 3mo, starting at 15mo of age. TauRD^ΔK^ mice received a daily dose of 20 mg/kg MB for 3mo and 14.5mo starting at 12mo or 1.5mo of age, respectively. Another group of TauRD^ΔK^ mice was treated with 40 mg/kg MB for 3mo, starting at 12mo of age. MB-treated groups were accompanied by groups of vehicle treated (H_2_O + saccharin) Tau transgenic littermates and by groups of wild-type littermates (MB or vehicle treated). Each group was composed of 6-11 age and gender matched animals.

### Behavior tasks

*Housing conditions: p*rior to behavior experiments, mice were single-housed under standard conditions with food, water and MB ad libitum in a room with inverted 12 h light/dark cycle to assure testing in the nocturnal phase of the animals.

*Open field test:* the open field consists of a 50 x 50 cm arena divided into 20 x 20 cm center, a 5 cm wall zone and a 10 cm border zone. Each mouse was placed into the center of the box and could freely explore the arena for 15 min while being tracked by a video system (Viewer II, Biobserve). The following parameters were analyzed: activity, distance moved, mean velocity, time spent in the center zone and distance to wall. Activity was defined as amount of active time (%) during the duration of stay, in which the mouse’s movement speed exceeded the activity threshold. The activity threshold defines a certain velocity limit to distinguish active from inactive behavior (1 cm/s).

*Morris water maze (MWM):* before starting the MWM experiment, a 2 days pretraining protocol was conducted to habituate the mice to swimming and climbing onto a hidden platform. To avoid any interference with the MWM learning, the pretraining was performed in a different room and apparatus than used for the MWM. Spatial memory abilities were examined in the standard hidden-platform acquisition and retention version of the Morris water maze [48]. A 180 cm circular pool was filled with water opacified with non-toxic white paint (Biofa Primasol 3011). The tank was divided into four quadrants: target (T), right adjacent (R), opposite (O), and left adjacent (L). A 15 cm round platform was hidden 1 cm beneath the surface of the water at a fixed position in the center of the target quadrant. The pool was surrounded by landmarks attached to the walls to facilitate orientation. Each mouse performed 4 swimming trials per day (maximum duration 90s, 10 min inter-trial interval) for five consecutive days. The time required to locate the hidden platform (escape latency), path length and swimming speed were determined. On acquisition day 3, 4, 5, as well as 2 days after the end of acquisition, a probe trial was conducted without platform. Acquisition and probe trials were recorded and analyzed by the Viewer II video tracking system (Biobserve).

### Biochemical analysis of brain tissue

Sarcosyl-extraction, total protein preparation and western blots were performed as described previously [14,15]. Depending on the 1^st^ antibody, 2-20 μg of total protein or 3 μl of sarcosyl extraction lysates from brain tissue were loaded for the detection with pan-Tau antibody K9JA (1:20000, Dako A-0024), the human Tau specific antibody TauY9 (1:2000, Enzo), phospho-Tau antibodies 12E8 (pSer262/pSer356, 1:500, Elan), PHF1 (pSer396/pSer404, 1:500, gift from Dr. P. Davies) and antibodies against synaptophysin (1:20000, Sigma), synapsin1 (1:2000, Novus Biologicals), PSD95 (1:2000, Dianova), Beclin (1:10000, Santa Cruz), HSC70 (1:10000, Abcam), lamp2a (1:2000, Abcam), PSMD13 (1:2000, Abcam). Blots were normalized by the concentration of β-actin (1:20000, Sigma).

### Histology

Immunohistochemistry was performed on 5 μm paraffin sections as described [14,15]. The following antibodies were used for light microscopy: MC1 (conformational epitope, aa 5-15 + 312-322, 1:10, gift of Dr. P. Davies, Albert Einstein College, NY) and AT180 (pThr231/pSer235, 1:1000, Pierce). Secondary antibodies as well as the avidin-biotinylated peroxidase complex were provided by the Vectastain Universal Elite ABC kit (Vector laboratories) and DAB (Dako) was used to visualize the antibody labeling. Gallyas silver impregnation was performed as described [14,15]. Immunofluorescence: the primary mouse monoclonal OXPHOS antibody cocktail (1:250, MitoScience) followed by a secondary anti-mouse DyLight 650 antibody (1:500, Thermo Fisher Scientific) was applied. Nuclei counterstain was performed with Syto13 (1:4000, Life Technologies GmbH). Stainings imaged by a LSM510 Meta confocal microscope (Zeiss) using lasers, beam splitters, and filters according to the fluorophores.

### Statistics

Open field test: MB-treated, untreated and control groups were compared by one-way ANOVA with post hoc Newman-Keuls multiple comparison test. Morris water maze: MB-treated, untreated and control groups were compared by two-way repeated ANOVA followed by a post hoc Fisher LSD multiple comparison test. Asterisks indicate differences between treated and untreated Tau^ΔK^ mice (MWM acquisition). For analysis of probe trials a two-tailed t-test against chance level (25%) or a one-way ANOVA with post-hoc Newman-Keuls multiple comparison test was done. Protein amounts were compared by one way ANOVA with post hoc Newman-Keuls multiple comparison test or by an unpaired two-tailed t-test. Numbers of samples are indicated in figure legends. All data are presented as group mean values with standard error of mean (SEM), the accepted level of significance was p < 0.05. Statistical comparisons were performed using STATISTICA 10.0 software (StatSoft), graphs were designed using Prism 5.0 software (GraphPad). *: p < 0.05, **: p < 0.01, ***: p < 0.001.

## Results

### Characteristics of pro-aggregant Tau transgenic mice

Inducible mice with constant expression of full-length pro-aggregant Tau^ΔK^ or repeat domain pro-aggregant TauRD^ΔK^ develop a progressive neuropathology including prominent cognitive deficits. Importantly, cognitive deficits as well as synaptic impairments recover after switching off the expression of human Tau [14,15], demonstrating that Tau-induced pathology can be reversed in principle. These studies provide the rationale for treatment of pro-aggregant mice using Tau-directed drugs.

From 3 months (mo) of age onwards (or after 3mo of Tau expression), repeat domain TauRD^ΔK^ mice show a pronounced neuropathology, especially in terms of Tau aggregation and neuronal death. By comparison, the brain pathology is much less pronounced in full-length Tau^ΔK^ mice. These mice develop a pre-tangle pathology indicated by the conformation-dependent antibody MC1 [49] but lack a silver-positive NFT pathology and neuronal loss in the hippocampus. The difference is consistent with the fact that TauRD^ΔK^ lacks the flanking regions (Additional file 1: Figure S1), which leads to a higher β-propensity, causing efficient aggregation and a stronger neurotoxicity than full-length Tau^ΔK^ at comparable expression levels. Note that TauRD^ΔK^ cannot react with antibody MC1, since it lacks part of the epitope; the same holds for other diagnostic antibodies (e.g. AT8) with epitopes outside the repeat domain. Therefore, the appearance of MC1 or AT8 reactivity in TauRD^ΔK^ mice originates from endogenous mouse Tau, which has been transformed to a pathological state, triggered by exogenous TauRD^ΔK^. By contrast, the epitope of antibody 12E8 (pS262 + pS356) lies within the repeats and is present in all Tau variants.

Due to the specific design of the transgenic DNA the expression of human Tau^ΔK^ and TauRD^ΔK^ can be deduced from luciferase activity by bioluminescence imaging (BLI) in living mice. This opens the possibility to preselect animals with comparable Tau expression prior to experiments in order to reduce inter-individual variations, which is important since Tau levels influence the severity of pathology [50].

### Pharmacokinetic properties of MB in mice

To check bioavailability and lifetime of MB in mice, MB levels in plasma and brain were measured after intravenous (i.v.) application of 30 mg/kg MB or oral application of 45 mg/kg MB (Additional file 2: Figure S2) in wild-type C57BL/6 mice (n = 3 per time point). MB levels were evaluated by quantification of tetramethylthioninium-ion (TMT-ion) concentrations using liquid chromatography - mass spectrometry (LC-MS/MS). TMT-ion equals MB without chloride and 3*H_2_O.

After i.v. administration, peak concentrations in plasma and brain were observed at 5 to 15 min post application and pharmacokinetic analyses over 24 h revealed MB half-lives of t_1/2_ = 4.4 h in plasma and t_1/2_ = 3.0 h in brain (Additional file 2: Figure S2a). Intravenous application of MB led to a reduced general condition and apathy of the mice, which may point to an acute toxic effect of the bolus. In contrast, oral administration of MB was well-tolerated and led to peak concentrations in plasma and brain within the first 2 hours post application. Pharmacokinetic analyses over 24 h exhibited MB half-lives of t_1/2_ = 4.7 h in plasma and t_1/2_ = 30.6 h in brain. The oral bioavailability was determined at ~18% (Additional file 2: Figure S2b). Furthermore, the results show a concentration of MB inside the brain, with ~30-fold and ~60-fold higher MB levels in brain tissue compared to plasma at 24 h post i.v. or oral application, respectively.

### MB application strategies

**Figure 1:**
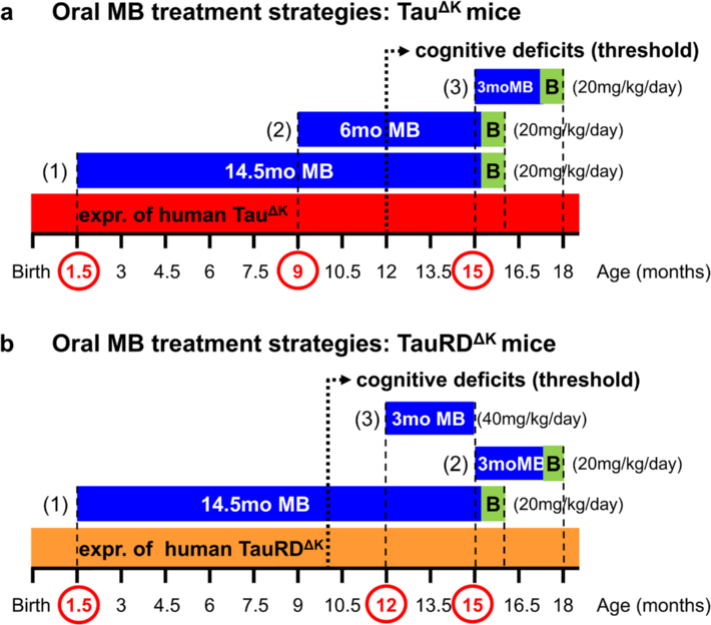
Oral MB treatment strategies of mice. **(a)** Full-lengthTau^ΔK^ mice received MB for 14.5mo starting at 1.5mo of age, before onset of Tau pathological changes and cognitive decline (protocol 1, 1.5 mo Tau pathology, 14.5 mo preventive MB, 20 mg/kg), for 6mo starting at 9mo of age, shortly before the onset of cognitive impairments (protocol 2, 9 mo Tau pathology, 6 mo preventive MB, 20 mg/kg) and for 3mo starting at 15mo of age, at a time point, when learning and memory deficits are already present (protocol 3, 15 mo Tau pathology, 3 mo therapeutic MB, 20 mg/kg). The constant expression of Tau^ΔK^ throughout the entire life-span in the absence of doxycycline is depicted as red bar. The onset of progressive cognitive failure starting ~12 months of age is represented by the dotted arrow. **(b)** TauRD^ΔK^ received MB for 14.5mo starting at the age of 1.5mo, before onset of Tau pathology and cognitive impairment (protocol 1, 1.5 mo Tau pathology, 14.5 mo preventive MB, 20 mg/kg) and for 3mo starting at 15mo of age, after the onset of cognitive decline (protocol 2, 15mo Tau pathology, 3mo therapeutic MB, 20 mg/kg). An excessive MB dose of 40 mg/kg was applied for 3mo at the age of 12-15mo (protocol 3, excessive MB, 40 mg/kg). The constant expression of TauRD^ΔK^ throughout life-span in the absence of doxycycline is depicted as orange bar. The onset of cognitive deficits starting ~10mo of age is represented by the dotted arrow. Periods and initiation of MB treatment are indicated by blue bars and red circles on the time axis, including period of behavioral testing during the final month (green box). The daily MB dose (20 or 40 mg/kg) for each treatment protocol is given in brackets. MB was administered via the drinking water. MB: methylene blue; mo: months; B: behavior tests; expr.: expression.

The goal of the study was to optimize MB treatment conditions in order to get the maximum beneficial effect on learning and memory performance of Tau transgenic mice. To this end we followed different treatment strategies (Figure 1). The major difference between the various treatment protocols is the initial time point of intervention, namely before or after onset of cognitive impairments, which start typically at ~12mo of age in Tau^ΔK^ mice and at ~10mo of age in TauRD^ΔK^ mice. The key question was, how much Tau pathology can be allowed while still preventing cognitive decline by MB treatment.

#### MB application strategies for pro-aggregant full-length Tau^ΔK^ mice

In preventive treatment protocol (1), MB application was initiated at 1.5mo of age and continued for 14.5mo beyond the anticipated onset of cognitive deficits typically around ~12mo of age in this mouse strain. The aim was to target Tau-induced changes very early on, before any neuropathological and cognitive changes occur (Figure 1a, preventive MB for 14.5mo).

In preventive treatment protocol (2), MB administration was initiated at 9mo of age, a time point ~3mo before the expected onset of cognitive impairment but with presence of early Tau^ΔK^-induced pathological changes such as conformational change, missorting and phosphorylation of Tau (Figure 1a, preventive MB for 6mo).

Finally, therapeutic treatment protocol (3) aimed to test whether therapeutic intervention by MB after onset of cognitive decline has the potential to rescue the existing neuropathology and phenotype (Figure 1a, therapeutic MB for 3mo). Therefore MB application was initiated at 15mo of age (~3mo after onset of cognitive impairments) and continued for 3mo. In this respect, treatment protocol (3) was closely analogous to the aforementioned Tau switch-off experiments, which resulted in a complete rescue of functional deficits [14,15]. All Tau^ΔK^ mice were treated using an oral daily dose of 20 mg/kg MB, which was administered via the drinking water.

#### MB application strategies for pro-aggregant repeat domain TauRD^ΔK^ mice

Similar to Tau^ΔK^ animals, TauRD^ΔK^ mice received MB (20 mg/kg) for 14.5mo, starting at 1.5mo of age, long before the appearance of Tau pathology and cognitive impairments (Figure 1b, protocol 1, preventive MB for 14.5mo).

In therapeutic treatment protocol (2), MB (20 mg/kg) application started at 15mo of age and continued for 3mo (Figure 1b, therapeutic MB for 3mo). At the age of 15mo, TauRD^ΔK^ mice exhibit strong neuropathological changes as described above and show severe learning and memory deficits.

Finally, TauRD^ΔK^ mice received an excessive MB dose of 40 mg/kg for 3mo at the age of 12-15mo to further evaluate the effect of MB on TauRD^ΔK^ aggregation (Figure 1b, therapeutic protocol (3), excessive MB for 3mo).

After MB treatment, mice were tested for their general motor and exploration behavior using an open field test and for their spatial reference learning and memory performance in a Morris water maze (MWM) test. In the open field test, no differences between MB-treated and untreated pro-aggregant Tau mice or wild-type (WT) mice were observed for average velocity and distance covered throughout all experimental setups. In addition swimming speed did not differ between these groups in the MWM test. Thus, we exclude that motor deficiencies of pro-aggregant Tau mice account for the differences detected in behavioral readouts (see below). This issue is of concern since some published Tau-transgenic mice developed motor deficits caused by transgene expression in the spinal cord due to Thy-1.2 or PrP promoters [11,13], which is avoided here by the use of the CaMKIIα promoter.

### MB treatment of pro-aggregant full-length Tau^ΔK^ mice

#### Preventive treatment starting 11mo before cognitive decline

In this protocol, MB treatment started shortly after birth and long before the expected onset of cognitive decline (~12mo), and continued beyond this for a total of 14.5mo (including the final month of behavioral testing, Figure 1a).

**Figure 2:**
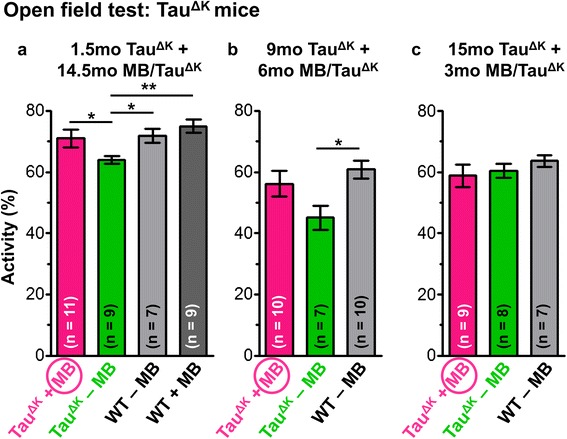
Exploration behavior of MB-treated Tau^ΔK^ mice. Total activity (%) within 15 min is analyzed by an open field test after various MB treatment periods. **(a)** Preventive MB treatment for **(a)** 14.5mo and for **(b)** 6mo results in preservation of exploration behavior of Tau^ΔK^ mice similar to wild-type (WT) animals, whereas untreated Tau^ΔK^ mice show a significantly reduced activity. By contrast no group-differences are observed after therapeutic MB treatment for 3mo **(c)**. Bars represent mean values ± SEM. Statistics: one-way analysis of variances with post-hoc Newman-Keuls multiple comparisons test. Asterisks indicate significant differences between groups; *: p < 0.05; **: p < 0.01; mo: months.

Regarding open field activity, the 14.5mo MB treatment resulted in significant group differences, such that untreated Tau^ΔK^ mice were less active than WT animals or MB-treated Tau^ΔK^ mice (Figure 2a, p = 0.004, Additional file 3: Figure S3). In contrast, no group differences were observed for anxiety-related parameters (time in center, distance to wall), suggesting that preventive MB treatment did not have anxiolytic effects (Additional file 4: Figure S4).

**Figure 3:**
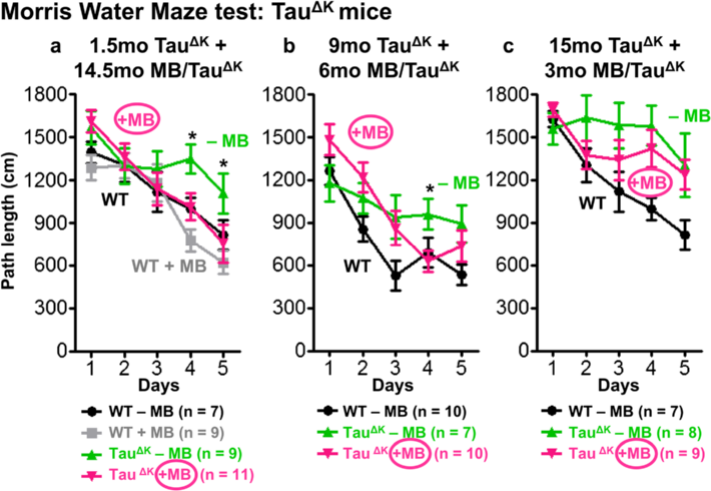
Spatial learning of MB-treated Tau^ΔK^ mice. Morris water maze (MWM) acquisition shows spatial learning abilities as indicated by path lengths (cm) of mice after MB application using different preventive and therapeutic treatment paradigms. Significant group differences are observed between untreated Tau^ΔK^ mice and WT animals throughout all experiments, demonstrating impaired learning abilities upon expression of pro-aggregant Tau **(a-c)**. By contrast Tau^ΔK^ mice treated with MB for 14.5mo **(a)** and 6mo **(b)** behave comparable to controls indicating preservation of cognitive functions. Short-term MB treatment for 3mo has no beneficial impact on the learning performance of Tau^ΔK^ mice **(c)**. Data shows mean path length ± SEM. Statistics: two-way repeated measure analysis of variances with post hoc Fishers LSD multiple comparisons test. Asterisks indicate differences between MB-treated and untreated Tau^ΔK^ mice; *: p < 0.05; n: number of mice.

During MWM acquisition, WT mice and MB-treated pro-aggregant full-length Tau^ΔK^ mice showed a superior learning performance over untreated Tau^ΔK^ mice (WT vs. Tau^ΔK^ - MB: p = 0.025). Importantly, MB-treated Tau^ΔK^ mice demonstrated a similar learning efficiency than WT animals (WT vs. Tau^ΔK^ + 14.5mo MB: p = 0.532) and performed significantly better than untreated Tau^ΔK^ at day 4 and day 5 (Tau^ΔK^ - MB vs. Tau^ΔK^ + 14.5mo MB; day 4: p = 0.025; day 5: p = 0.018) (Figure 3a). In probe trials, MB-treated Tau^ΔK^ mice exhibited a higher preference for the target quadrant and a more precise localization of the target platform position in comparison to untreated Tau^ΔK^ mice (Additional file 5: Figure S5). The results indicate a preservation of cognitive abilities upon preventive MB treatment initiated before the onset of Tau pathological changes and cognitive decline. Interestingly, WT mice receiving MB for 14.5mo, showed a slightly superior learning performance over untreated WT mice (Figure 3a), pointing towards an additional beneficial effect of MB on brain metabolism.

**Figure 4:**
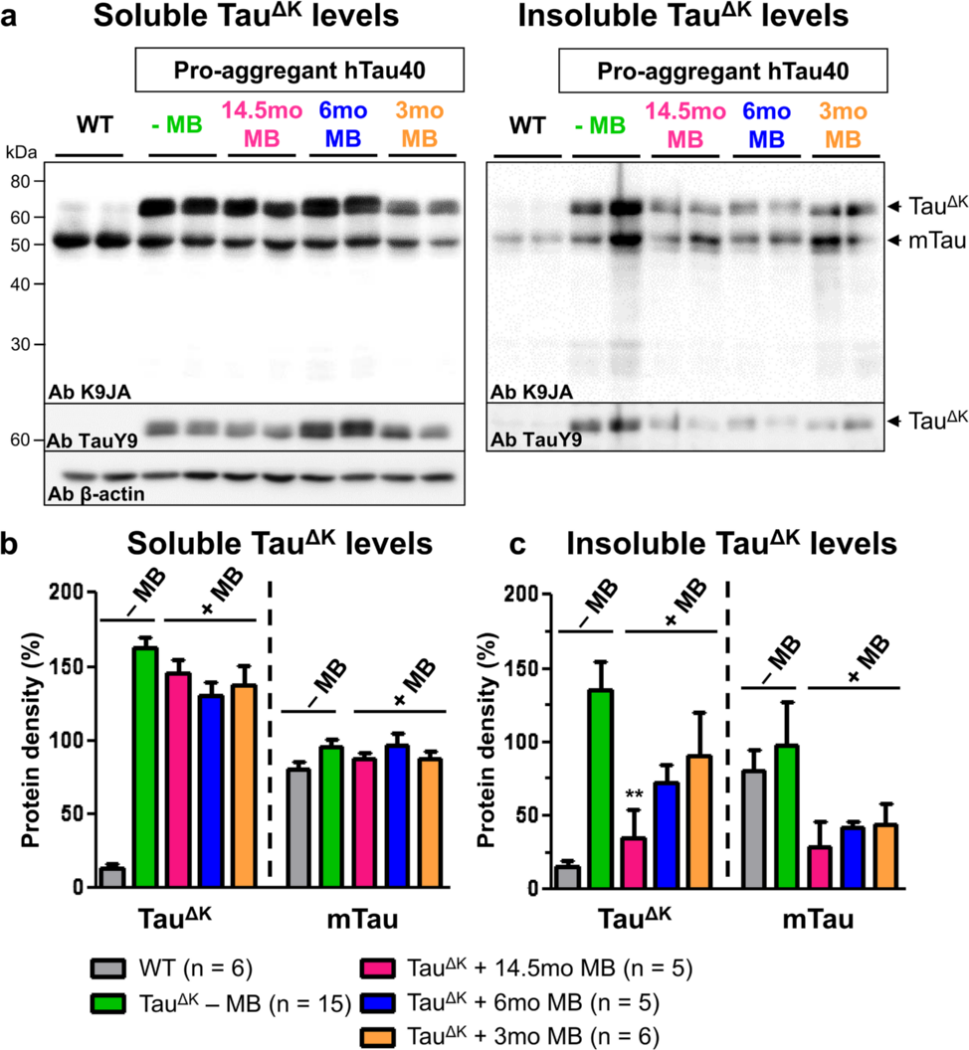
MB treatment reduces detergent insoluble Tau^ΔK^ and mouse Tau. Sarcosyl-extraction of soluble and insoluble Tau from cortex tissue. **(a)** Protein levels of soluble and insoluble Tau species in untreated Tau^ΔK^ mice compared to Tau^ΔK^ mice receiving MB for 14.5mo, 6mo and 3mo detected by the pan-Tau antibody K9JA and the human Tau specific antibody TauY9. MB administration reduces levels of detergent-insoluble human and mouse Tau. **(b)** Quantification of soluble Tau levels in cortex lysates shows a minor but non-significant decrease of soluble Tau species upon MB treatment. **(c)** Quantification of insoluble Tau levels in cortex lysates demonstrates a clear decrease of insoluble human and mouse Tau in MB-treated mice. Bars represent mean protein densities (%) ± SEM; statistics: one-way analysis of variances with post-hoc Newman-Keuls multiple comparisons test. Asterisks indicate significant differences in comparison to untreated Tau^ΔK^ mice; **: p < 0.01; Ab: antibody; mTau: mouse Tau; n, number of samples.

**Figure 5:**
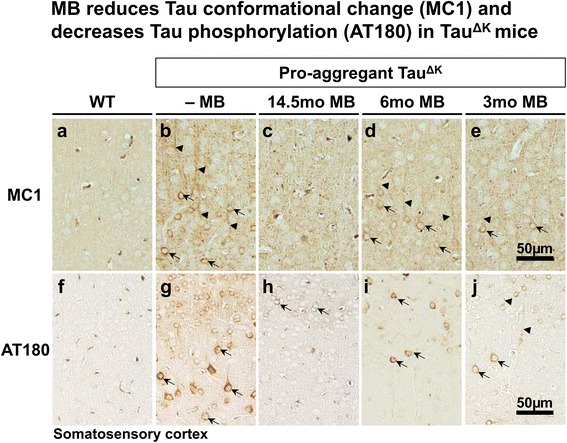
Histological analysis of conformationally changed and phosphorylated Tau in MB-treated mice. **(a-e)** MC1 immunoreactivity (epitope 5-15 + 312-322) indicates a pathological Tau conformation. MC1 positive neurons are prominent in somatosensory cortex (SSCx) of untreated Tau^ΔK^ mice with missorting of Tau to cell soma (arrows) and apical dendrites (arrowheads). In contrast MB treatment, especially preventive MB treatment for 14.5mo, clearly reduces MC1 immunoreactivity in Tau^ΔK^ mice. **(f-j)** Histological analysis of phosphorylated Tau using the AT180 antibody (dual phosphorylation epitope pThr231 + pSer235). Untreated Tau^ΔK^ mice display a massive mislocalization of phosphorylated Tau to cell soma (arrows) and apical dendrites (arrowheads) of SSCx neurons, whereas MB treatment diminishes the extent of AT180 phosphorylation. Scale bar: 50 μm.

**Figure 6:**
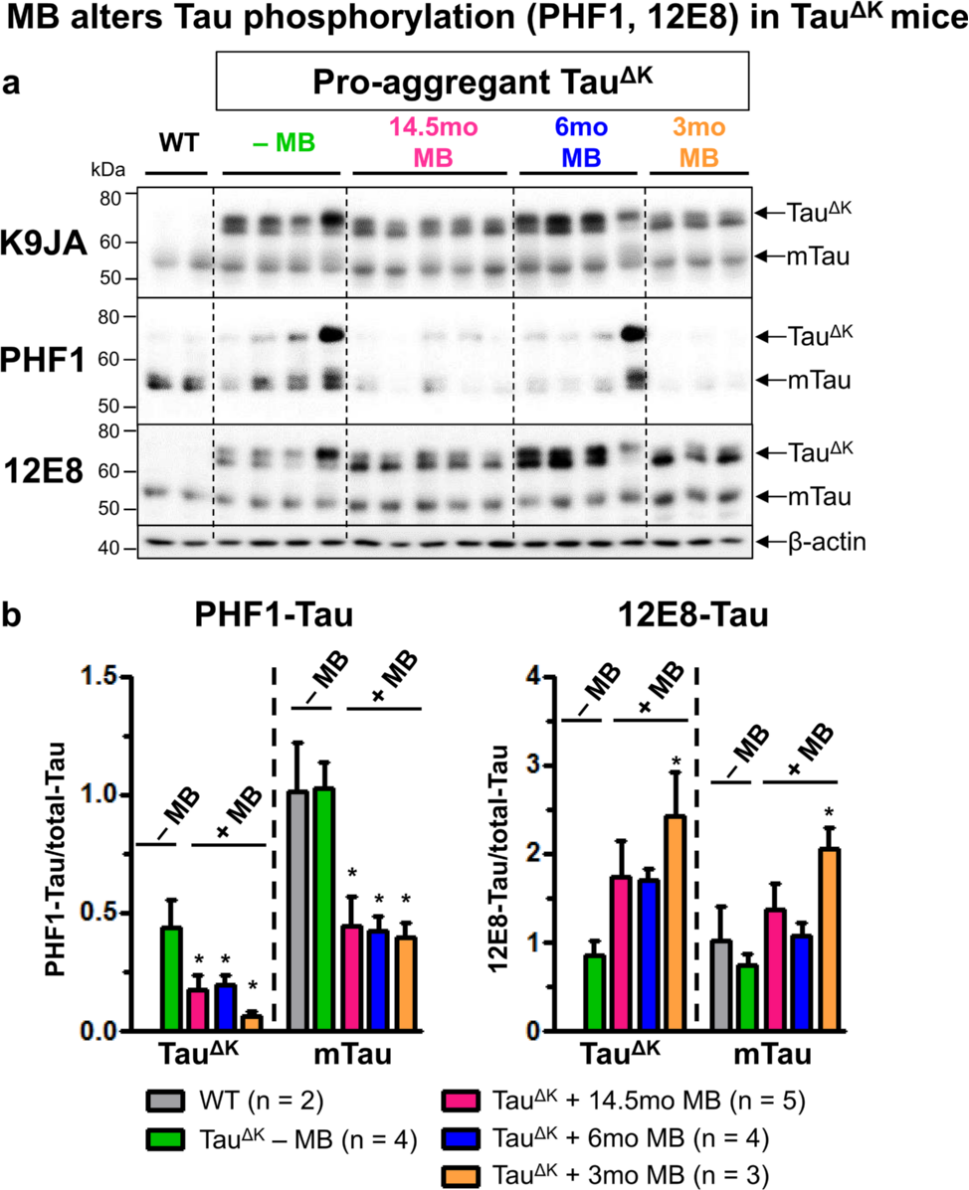
MB application alters levels of phosphorylated Tau. **(a)** Levels of total Tau (pan-Tau antibody K9JA), PHF1 phosphorylated (epitope pSer396/pSer404) and 12E8 (epitope pSer262/pSer356) phosphorylated Tau in cortex homogenates of MB-treated and untreated Tau^ΔK^ mice. MB administration for 14.5mo, 6mo and 3mo results in a decrease of PHF1 phospho-Tau as compared to untreated Tau^ΔK^ mice. By contrast an increase of 12E8 phospho-Tau inside the repeat domain of Tau is observed after MB treatment, indicating detachment from microtubules. **(b)** Determination of relative protein densities by quantification of **(a)**. Levels of phosphorylated Tau were normalized to total Tau levels. Bars represent mean values ± SEM. Statistics: one-way analysis of variances with post-hoc Newman-Keuls multiple comparisons test. Asterisks indicate significant differences in comparison to untreated Tau^ΔK^ mice; *: p < 0.05; n, number of samples.

MB application for 14.5mo (started at 1.5mo of age) reduced insoluble Tau^ΔK^ and endogenous mouse Tau, compared with untreated Tau^ΔK^ mice (Figure 4). Similarly, a decrease of conformationally-changed Tau (epitope MC1, 5-15 + 312-322, Figure 5, Additional file 6: Figure S6) and a reduction of phosphorylated Tau (epitope AT180, pThr231 + pSer235 and epitope PHF1, pSer396 + pSer404) was observed after 14.5mo MB treatment (Figure 5, Figure 6, Additional file 6: Figure S6). By contrast, Tau phosphorylated at the KXGS motifs inside the repeat domain (epitope 12E8, pSer262 + pSer356) was increased in MB-treated animals (Figure 6). Note that Tau phosphorylation at the KXGS motifs causes detachment of Tau from microtubules, but also protects Tau against aggregation [51].

**Figure 7:**
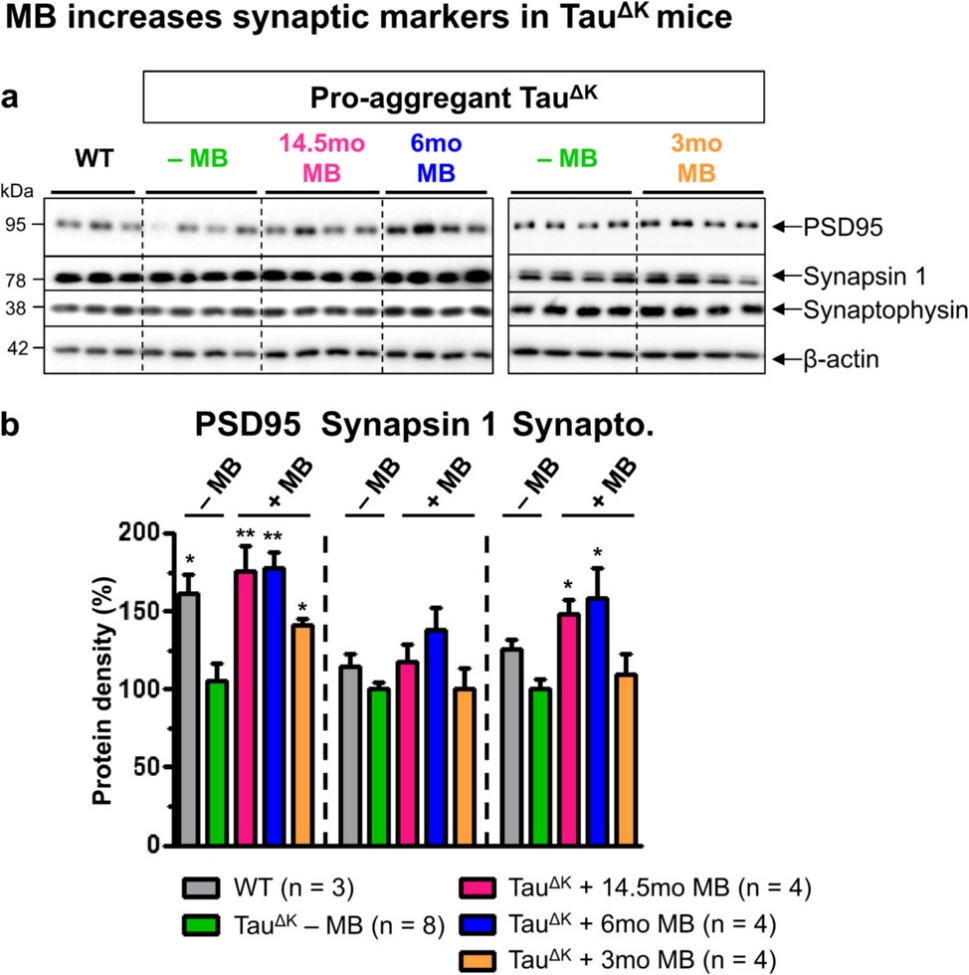
Recovery of synaptic marker proteins in hippocampus of MB-treated mice. **(a)** Levels of pre- and post-synaptic proteins synaptophysin, synapsin 1 and postsynaptic density 95 (PSD95) in hippocampus lysates of age-matched untreated and MB-treated Tau^ΔK^ mice. β-actin serves as loading control. **(b)** Determination of relative protein densities by quantification of **(a)**. Untreated Tau^ΔK^ mice show a constant decrease of synapsin 1, synaptophysin and PSD95 levels in comparison to WT, due to constant expression of pro-aggregant Tau^ΔK^. In contrast a recovery of pre- and postsynaptic markers is observed after preventive MB application for 14.5mo and 6mo, whereas therapeutic MB treatment for 3mo causes a partial rescue of post- but not of presynaptic markers. Protein levels were normalized to β-actin. Bars represent mean values ± SEM. Statistics: one-way analysis of variances with post-hoc Newman-Keuls multiple comparisons test. Asterisks indicate significant differences in comparison to untreated Tau^ΔK^ mice; *: p < 0.05; **: p < 0.01; n, number of samples.

Previously, we described a close relationship between expression of pro-aggregant Tau, synaptic failure inside the hippocampus formation and cognitive decline [14,15]. While untreated pro-aggregent Tau^ΔK^ mice showed a consistent reduction in pre- and postsynaptic proteins (i.e. synapsin 1, synaptophysin, PSD95), MB treatment for 14.5mo preserved pre- and postsynaptic protein levels (Figure 7), suggesting a protective effect of MB on the synaptic integrity which likely contributes to the preservation of cognition.

**Figure 8:**
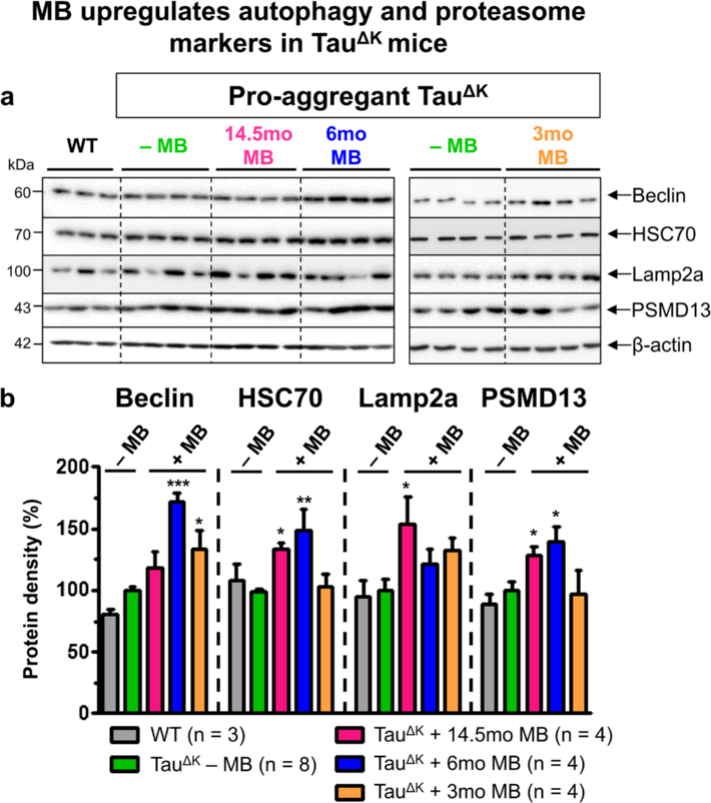
Preventive MB administration increases protein degradation. **(a)** Levels of autophagy- (beclin, HSC70), lysosome- (lamp2a), and proteasome-related (PSMD13) proteins in hippocampus lysates of age-matched untreated Tau^ΔK^ mice compared to MB-treated Tau^ΔK^ mice for 14.5mo, 6mo and 3mo. β-actin serves as loading control. **(b)** Quantification of **(a)**. While both preventive MB treatment strategies (14.5mo and 6mo MB) increase levels of beclin, HSC70, Lamp2a and PSMD13, therapeutic MB treatment for 3mo leads to a partial increase of beclin and Lamp2a whereas levels of HSC70 and PSMD13 remain unaffected in comparison to untreated Tau^ΔK^ mice. Protein levels were normalized to β-actin. Bars indicate mean values ± SEM. Statistics: one-way analysis of variances with post-hoc Newman-Keuls multiple comparisons test. Asterisks indicate significant differences in comparison to untreated Tau^ΔK^ mice; *: p < 0.05; **: p < 0.01; ***: p < 0.001; n, number of samples.

Clearance of proteins via autophagy is a major pathway to maintain neuronal homeostasis and health. Preventive MB treatment of Tau^ΔK^ mice for 14.5mo increased levels of beclin, heat shock cognate protein 70 (HSC70) and lysosome-associated membrane protein 2a (Lamp2a) (Figure 8), indicating an upregulation of autophagy. While beclin is involved in the initiation of the autophagosome formation during macroautophagy, HSC70 (a constitutively expressed molecular chaperone) and Lamp2a (lysosomal receptor) play a role in chaperone-mediated autophagy (CMA) of Tau (Wang et al., 2009). Another prominent pathway for protein degradation is the ubiquitin-proteasome system. As judged by an increased level of PSMD13 (regulatory subunit of the 26S proteasome), 14.5mo MB treatment enhanced proteasome function in comparison to WT and untreated Tau^ΔK^ mice (Figure 8). Taken together, the tendency of MB to preserve or enhance protein degradation may counteract the continuous accumulation of toxic Tau species and contribute to maintenance of protein homeostasis.

MB’s role as electron carrier and thus redox-cycling compound is widely discussed in the literature [52]. However, we did not detect obvious differences between MB-treated and untreated Tau^ΔK^ mice regarding mitochondria distribution and proteins of the electron transport chain (Additional file 7: Figure S7), indicating that MB did not alter this route of neuronal energy production.

In summary, if preventive treatment was started at an early age the cognitive abilities of Tau^ΔK^ mice were essentially preserved, well beyond the expected onset of decline and in spite of the continued expression of the toxic mutant full-length Tau^ΔK^.

#### Preventive treatment starting 3mo before cognitive decline

Preventive MB administration (protocol 2, started ~3mo before onset of cognitive decline and continued for 6mo) showed an increase of open field activity of Tau^ΔK^ mice in comparison to the reduced activity of untreated Tau^ΔK^ mice (Figure 2b, Additional file 3: Figure S3). No group differences concerning anxiety related parameters were observed (Additional file 4: Figure S4). In MWM acquisition, both untreated Tau^ΔK^ and 6mo MB-treated Tau^ΔK^ mice performed less well than WT mice (WT vs. Tau^ΔK^ - MB: p = 0.017; WT vs. Tau^ΔK^ + 6mo MB: p = 0.036, Figure 3b). However, Tau^ΔK^ mice with 6mo MB application showed a better learning performance compared to untreated Tau^ΔK^ on day 4 (Tau^ΔK^ - MB vs. Tau^ΔK^ + 6mo MB: p = 0.042, Figure 3b). In addition they were able to localize the target quadrant and platform position more precisely than untreated Tau^ΔK^ animals (Additional file 5: Figure S5), implying a minor beneficial effect of MB on cognition.

Neuropathological analysis revealed a reduction of insoluble Tau^ΔK^ and mouse Tau (Figure 4), a decrease of conformationally-changed Tau (MC1) and phosphorylated Tau (AT180, PHF1) after 6mo of MB administration (Figure 5, Figure 6, Additional file 6: Figure S6). In addition, synaptic and autophagy marker proteins as well as the proteasome marker PSMD13 were increased after 6mo MB (Figure 7, Figure 8) but mitochondria remained unaffected (Additional file 7: Figure S7).

Thus, if preventive treatment was started at adult age with progressive tau pathology but still before the anticipated onset of cognitive decline, cognitive abilities were protected to a certain extent, in spite of the continued expression of toxic Tau^ΔK^.

#### Therapeutic treatment starting 3mo after cognitive decline

Finally, we tested the therapeutic potential of MB to reverse cognitive deficits ~3mo after onset (Figure 1a, protocol 3). MB treatment for 3mo did not enhance open field activity of Tau^ΔK^ mice. Thus, treated Tau^ΔK^ mice behaved similarly to untreated Tau^ΔK^ animals (Figure 2c). WT mice showed a slight but non-significant increase in open field activity compared to transgenic animals, which was most pronounced within the first 5 minutes of the experiment (Additional file 3: Figure S3) and probably due to an advanced age of the mice during testing. Anxiety-related parameters were unaffected (Additional file 4: Figure S4).

An MWM test indicated a severe learning impairment of 3mo MB-treated Tau^ΔK^ mice similar to untreated Tau^ΔK^ mice and in contrast to WT animals, which improved consistently on each acquisition day (WT vs. Tau^ΔK^ - MB: p = 0.001; WT vs. Tau^ΔK^ + 3mo MB: p = 0.011, Figure 3c). In addition, MB-treated mice did not show any preference for the target quadrant (Additional file 5: Figure S5), underlining the persistence of cognitive deficits after 3mo MB.

Nevertheless, in spite of the failure in improving cognitive defects, MB treatment was still able to reduce sarcosyl-insoluble Tau, although to a minor extent in case of Tau^ΔK^ compared to preventive MB treatment paradigms (Figure 4). In addition, conformationally changed Tau (MC1) and phosphorylated Tau (AT180, PHF1) were clearly reduced after 3mo MB treatment (Figure 5, Figure 6, Additional file 6: Figure S6).

By contrast, the 3mo MB application was not able to reverse the decline of presynaptic markers synaptophysin and synapsin 1 and the protective effect on postsynaptic PSD95 was less pronounced than in preventive MB treatment protocols (Figure 7). These results point towards sustained synaptic malfunction, which may underlie cognitive impairment independently of the reversal of biochemical Tau parameters. In addition, 3mo MB treatment only partly affected protein degradation systems. Whereas an increase in beclin and Lamp2a levels was detected, levels of HSC70 and PSMD13 were comparable to untreated age-matched Tau^ΔK^ mice (Figure 8). As before, no effect on mitochondria was observed (Additional file 7: Figure S7).

### Preventive and therapeutic MB treatments of TauRD^ΔK^ mice

In contrast to full-length Tau^ΔK^ mice, animals expressing the repeat domain TauRD^ΔK^ develop a pronounced brain pathology in terms of Tau aggregation (neurofibrillary tangles) and neuronal loss and exhibit an earlier onset of cognitive decline ~10mo of age. This is consistent with the fast aggregation of the protein in vitro caused by the higher propensity for β-structure, and the absence of the N-and C-terminal flanking domains (Additional file 1: Figure S1). As in the case of full-length Tau^ΔK^, we tested different treatment protocols for TauRD^ΔK^ mice (Figure 1b).

We performed preventive MB treatment for 14.5 mo, starting at 1.5mo of age, long before onset of cognitive decline (Figure 1b, protocol 1), as well as therapeutic treatment for 3 mo, starting 5mo after onset of cognitive decline (at age 15 mo) (Figure 1b, protocol 2). Both treatments failed to decrease the level of insoluble Tau (exogenous and endogenous, Additional file 8: Figure S8) and did not protect synapses or induce protein degradation via autophagy (data not shown).

**Figure 9:**
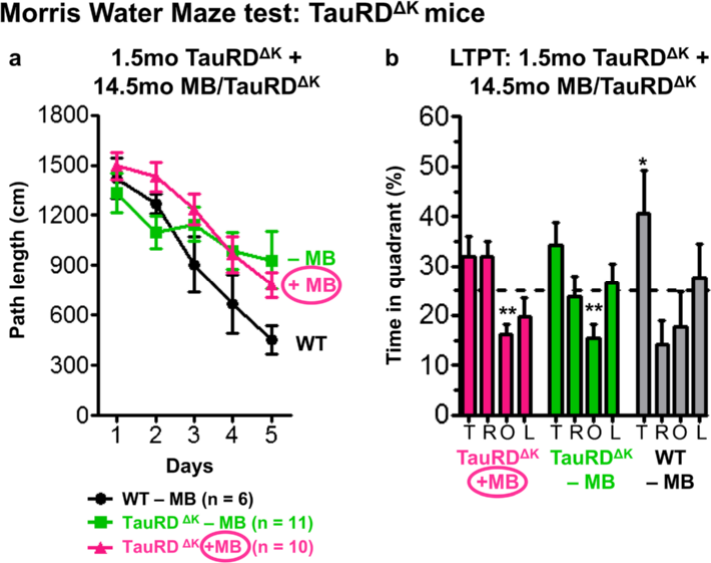
Preventive MB treatment does not rescue cognitive decline of TauRD^ΔK^ mice. MWM test shows cognitive abilities of TauRD^ΔK^ mice after preventive MB application for 14.5mo. **(a)** MB-treated as well as untreated TauRD^ΔK^ animals show impaired learning abilities similar to untreated TauRD^ΔK^ littermates, as indicated by increased path lengths to reach the hidden platform in MWM acquisition in comparison to WT. Data shows mean path length ± SEM. **(b)** Preventive MB treatment for 14.5mo does not result in a higher preference of the target quadrant as compared to untreated TauRD^ΔK^ mice and controls. Bars represent mean values ± SEM. Statistics: two-tailed one sample t-test against chance level of 25%; *: p < 0.05; **: p < 0.01; n = number of animals; T: target quadrant; R: right quadrant; O: opposite quadrant; L: left quadrant; LTPT: long-term probe trial.

Surprisingly, even long-term preventive treatment of TauRD^ΔK^ mice (14.5mo) failed to reverse Tau-induced cognitive deficits (Figure 9). MB-treated TauRD^ΔK^ mice did not learn the location of the hidden platform, similar to untreated TauRD^ΔK^ animals (Figure 9a), and did not show a significant preference for the target quadrant in subsequent probe trials during MWM (Figure 9b). Thus, 20 mg/kg/day MB was not sufficient to suppress Tau aggregation and subsequent neurotoxic processes initiated by TauRD^ΔK^.

**Figure 10:**
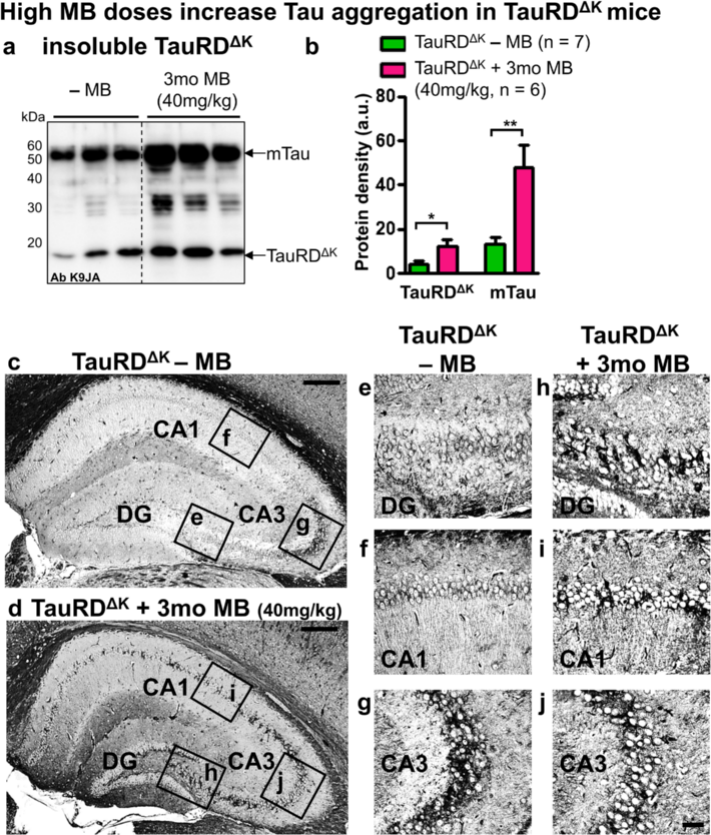
High MB-doses increase Tau aggregation. **(a)** Sarcosyl-extraction of insoluble Tau from cortex tissue of TauRD^ΔK^ mice. MB application using a daily dose of 40 mg/kg for 3mo results in an increase of sarcosyl-insoluble human TauRD^ΔK^ and endogenous mouse Tau (antibody K9JA). **(b)** Quantification of **(a)** shows a ~2-3 fold increase of insoluble Tau after high-dose MB treatment for 3mo. Bars represent mean values ± SEM. Statistics: two-tailed t-test; *: p < 0.05; **: p < 0.01; n, number of samples. **(c, d)** Gallyas silver staining of Tau aggregates shows an increase of tangle-positive cells inside the hippocampus formation of MB-treated TauRD^ΔK^ mice (40 mg/kg for 3mo). Scale bar: 200 μm. Boxed areas indicate close-ups presented in **(e-j)**. Higher magnification of dentate gyrus (DG), CA3 and CA1 hippocampal areas of untreated TauRD^ΔK^ mice **(e-g)** in comparison to corresponding areas of MB-treated TauRD^ΔK^ mice **(h-j)**. CA3: *region III cornus ammonis*; CA1: *region I cornus ammonis.* Scale bar: 40 μm.

To investigate whether higher doses of MB would influence TauRD^ΔK^ pathology, the mice were treated with an excessive dose of MB (40 mg/kg/day MB) for 3mo (Figure 1b, protocol 3, ~12-15mo of age, after cognitive decline). This actually resulted in an increase of aggregated Tau species in the hippocampus of TauRD^ΔK^ mice as detected by sarcosyl extraction and Gallyas silver staining (Figure 10). However, phosphorylated Tau was unaltered and autophagy partly reduced (data not shown). This detrimental effect of high doses MB was further confirmed by cell culture experiments. Inducible N2a cells expressing pro-aggregant TauRD^ΔK^ [53] accumulated aggregated Tau species in response to high concentrations of MB (25-100nM) in a dose-dependent manner (Additional file 9: Figure S9). Especially MB concentrations >100nM caused a massive loss of N2a cells, indicating a toxic gain of function in cells.

## Discussion

In this study we analyzed different protocols to determine optimal conditions for the preservation of cognitive abilities in pro-aggregant Tau transgenic mice treated with the Tau-aggregation inhibitor Methylene Blue (MB). Pro-aggregant full-length human Tau^ΔK^ or repeat domain TauRD^ΔK^ was expressed in inducible transgenic mice from birth onwards, leading to onset of cognitive decline at ~12mo or 10mo, respectively. The questions to be tested were: can treatment before the expected onset prevent the cognitive decline? Can treatment after the onset rescue the decline?

The results show that (1) MB preserves cognition in full-length pro-aggregant Tau^ΔK^ mice upon preventive treatment, provided that the intervention starts before the onset of behavioral deficits, whereas treatment initiated after onset of cognitive decline does not rescue. In addition, preventive MB application starting at young age (~1.5mo) has a greater impact on the cognitive performance than preventive MB application initiated at advanced age (~9mo) but shortly before onset of cognitive impairments. (2) MB reduces Tau aggregation, Tau phosphorylation and pathological Tau conformation in Tau^ΔK^ mice. (3) MB protects synapses and enhances protein degradation by autophagy but has no obvious effect on mitochondria in Tau^ΔK^ mice. (4) MB fails to prevent cognitive deficits in pro-aggregant repeat domain TauRD^ΔK^ mice, even when applied shortly after birth, presumably because the high aggregation propensity of TauRD^ΔK^ overwhelms the protective actions of the drug. (5) MB administered at high doses increases Tau aggregation rather than counteracting it.

### Absorption and distribution of MB in mice

Methylene blue was well tolerated in wild-type mice after oral application and reached plasma and brain concentrations beyond the detection level over a time course of 24 h. At 24 h post application, MB brain levels were ~30-60-fold higher as compared to MB plasma levels after i.v. and oral administration, respectively. The data demonstrates that MB is well absorbed, readily crosses the blood-brain barrier and concentrates in the nervous tissue, confirming earlier reports of MB pharmacokinetics in mice [39]. In good agreement to previous studies analyzing pharmacokinetic profiles of MB in humans [54,55], MB peak-levels in plasma and brain were observed 1-2 h after oral intake. In this study, MB plasma half-life times of 4-5 h and brain half-life times ~3 h or ~30 h were calculated after i.v. or oral MB application. In comparison, human MB plasma half-life times in the range of ~5-6 h [2,54] to ~18 h [55] were determined, whereas half-life times in human brains are not available. In addition the total bioavailability of MB seems to be lower in mice (~20%) in comparison to humans (~70%, [55]). These variations may arise from different absorption and distribution properties of MB in rodents, which further complicate a direct comparison of human and rodent MB dosing despite FDA surface area conversion tables [56]. Furthermore, pharmacokinetic analyses presented in this study reflect absorption and distribution of MB in wild-type mice, which may slightly differ in aged Tau^ΔK^ and TauRD^ΔK^ animals. Since Tau transgenic mice develop an age-dependent Tau neuropathology, alterations of the blood-brain barrier permeability cannot be excluded.

### MB’s therapeutic potential depends on the time point of intervention and Tau aggregation

Tau aggregation requires detachment of Tau from microtubules, which is increased by Tau phosphorylation inside the repeat domain [57]. Especially disease- and stress-related conditions induce mislocalization of unbound Tau into postsynaptic sites, which is connected to increased phosphorylation and aggregation of Tau and impaired microtubule interactions [58,59]. In further consequence, accumulation of phosphorylated Tau in dendritic spines causes functional impairments, which finally lead to the loss of synapses [60].

The process of Tau aggregation includes several stages: the repeat-domain of Tau contains motifs with increased tendency to form β-structures, which function as aggregation-prone templates and are enhanced by mutations such as ΔK280 [61]. In seeded assembly, Tau nuclei trigger further aggregation by interaction with other Tau repeat domains from both exogenous human and endogenous mouse Tau, resulting in the co-assembly into neurofibrillary tangles.

After switch-off, the synthesis of new pro-aggregant β-structure seeds is blocked, while existing aggregates (containing human and mouse Tau) are gradually cleared by the intracellular machinery. However, endogenous mouse Tau is still continuously expressed and can be incorporated into existing seeds and polymers. Thus the remaining aggregates mainly consist of mouse Tau, which is less toxic to synapses [14].

According to this scheme, removing or blocking amyloidogenic Tau counteracts Tau pathology. As a consequence of this “genetic treatment”, cognitive impairment can be rescued by an overall elimination of toxic Tau, even after cognitive decline has set in. This holds for both models, pro-aggregant full length Tau (Tau^ΔK^) and repeat domain Tau (TauRD^ΔK^) [14,15]. By contrast, aggregation inhibitors such as MB interfere with the aggregation process but do not affect de novo synthesis of pro-aggregant toxic Tau. This allows the formation of new β-structured seeds, which continue to aggregate. This would explain why MB can prevent pathology if administered at an early time point (because this keeps the seeds for aggregation below a critical threshold) but is inefficient if administered too late.

Several studies have confirmed the efficacy of MB in models of tauopathy when administered at early time points. A recovery of Tau-induced pathology and related locomotor phenotype was reported for a C. elegans model of tauopathy [35]. In Tau transgenic mice improvements of learning and memory behavior as a consequence of MB application was reported for Tau-P301S mice [62] and rTg4510 (Tau-P301L) mice [39]. In the successful treatment protocol used by Stack et al. (2014), MB (4 mg/kg/day) was administered for 10mo, starting at 1mo of age. Typically, Tau-P301S mice (line PS19) develop cognitive deficits ~6mo of age [8,63]. Thus, MB treatment was initiated ~5mo before the onset of cognitive impairment, and therefore this can be considered as a preventive strategy. O’Leary et al. (2010) reported an improved cognitive performance of rTg4510 mice after 3mo of MB treatment (10 mg/kg/day) starting at 3mo of age. Both studies are consistent with our results, which demonstrate a beneficial effect of MB in Tau^ΔK^ mice, when administered using preventive application strategies.

The data underscores the need of early biomarkers (e.g. from cerebrospinal fluid, brain imaging, etc.), which would identify asymptomatic patients at a point where toxic Tau aggregates have not yet reached a critical level, so that aggregation inhibitors have a chance to keep them from becoming super-critical [64,65].

### MB interference in Tau^ΔK^ and TauRD^ΔK^ mice

Why is MB able to counteract the pathology of full-length Tau^ΔK^ but not that of repeat domain TauRD^ΔK^? Removal of the flanking domains outside the repeats of Tau is known to enhance Tau aggregation [53,66,67] because this exposes the pro-aggregant hexapeptide motifs of Tau which are normally shielded by the N- and C-terminal domains [68]. Tau aggregates of repeat domain TauRD^ΔK^ mice are positive for the amyloid marker thioflavin S, and fibers can be visualized by electron microscopy. By contrast, mice expressing full-length Tau^ΔK^ lack these characteristics because this protein aggregates more slowly, has a low expression level, and does not reach the stage of mature Gallyas-positive tangles inside the hippocampus. Nevertheless, the “pre-tangles” contain smaller aggregates and oligomers, which are sufficient to cause synaptic decay and cognitive decline, with later onset than mice expressing the repeat domain.

**Figure 11:**
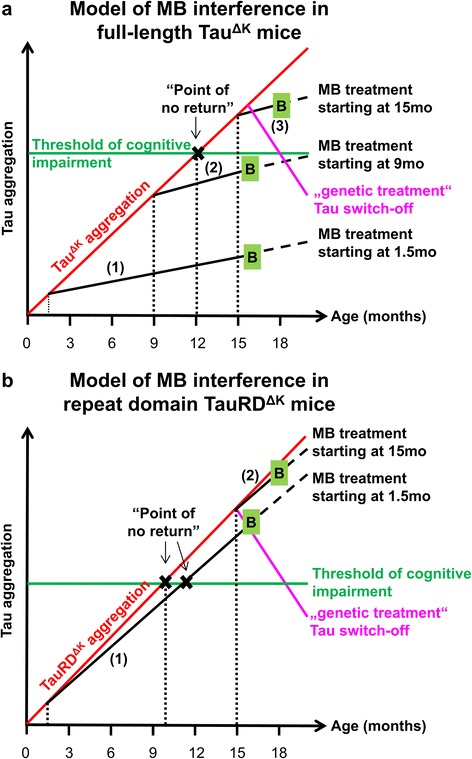
Models of MB interference during disease progression in full-length Tau^ΔK^ and repeat domain TauRD^ΔK^ mice. **(a)** In Tau^ΔK^ mice unaltered Tau aggregation due to continuous expression and accumulation of toxic Tau reaches the threshold of cognitive impairment at the age of ~12mo. Once the “point of no return” is passed, Tau^ΔK^ mice exhibit cognitive impairments and synaptic decay [15]. Preventive application of MB, starting before the critical threshold is reached, efficiently interferes with Tau^ΔK^ aggregation. Thus, the disease progression is slowed down and the onset of cognitive decline postponed. However, therapeutic MB application initiated at 15mo of age, beyond the “point of no return” and after the onset of cognitive impairment, is not able to rescue the cognitive phenotype. By contrast, “genetic treatment” by Tau^ΔK^ switch-off can reduce aggregation and reverse the cognitive decline. **(b)** TauRD^ΔK^ exhibits stronger aggregation properties than Tau^ΔK^, due to a higher β-propensity. Thus, TauRD^ΔK^ mice show a faster disease progression with onset of cognitive decline ~10mo of age [14]. MB’s anti-aggregation properties are not strong enough to efficiently retard the aggregation process, even if applied at early age (1.5mo). Consequently, the “point of no return” is barely delayed in TauRD^ΔK^ mice, and therapeutic treatment is ineffective. Similar to Tau^ΔK^, genetic treatment by TauRD^ΔK^ switch off is still able to reverse the cognitive phenotype even after onset of cognitive impairment. Red line: unaltered progression of Tau aggregation; green line: threshold of cognitive impairment; black X: “point of no return”; solid black lines: MB treatment periods of mice; magenta lines: “genetic treatment” by switch off of Tau expression; B, green boxes: behavior tests and termination of experiments; dotted black lines: assumed further progression of Tau aggregation; (1, 2, 3): MB treatment protocols according to Figure 1; mo: months.

Figure 11 illustrates a hypothetical progression model for both full-length Tau^ΔK^ and repeat domain TauRD^ΔK^ mice. In both models, progressive Tau aggregation (red line) due to continuous Tau expression proceeds to a threshold indicating the onset of cognitive impairment (“point of no return”, marked by X, Figure 11a,b). In the case of pro-aggregant full-length Tau^ΔK^ mice, the “point of no return” is reached at the age of ~12mo (Figure 11a). The slope of the aggregation can be reduced by MB (black lines corresponding to treatment protocols 1, 2, 3). If this takes place before the “point of no return”, cognitive impairment is delayed to a much later age (protocols 1, 2). If treatment starts after the “point of no return”, cognitive decline cannot be rescued, in spite of the slower aggregation rate (protocol 3).

The principle in case of TauRD^ΔK^ is similar, with the major difference that Tau aggregation is stronger and leads to a faster disease progression with earlier onset of cognitive deficits ~10mo of age (Figure 11b). Here the anti-aggregation potential of MB is not strong enough to retard Tau aggregation significantly and postpone the onset of cognitive impairment, even if treatment is started at early age (protocol 1). In contrast to treatment with an aggregation inhibitor, “genetic treatment” by switching-off Tau^ΔK^ or TauRD^ΔK^ reverses the slope (due to ongoing protein degradation) so that cognitive decline can be rescued (Figure 11a + b, magenta line, [14,15,69]).

### Direct interference of MB and Tau

MB is a redox-recycling agent and interacts with exposed cysteines, nucleotide-binding proteins and others; this may explain its many uses in medicine [70]. With regard to Tau, MB acts as an aggregation inhibitor [24,25,30] by a mechanism based on the oxidation of cysteine residues C291 and C322. They lie within the Tau repeat domain and affect the conformations and interactions leading to aggregation [71,72]. In addition, MB has been reported to lower the level of Tau by interacting with the chaperone hsp70 [73].

Beside MB (IC_50_: 1.9 μM, [24]), other potent Tau aggregation inhibitors have been identified (reviewed in [21,74-76]). Especially rhodanines (e.g. bb14, IC_50_ = 0.67 μM) and phenylthiazolyl-hydrazides (PTHs, i.e. BSc3094, IC_50_ = 1.6 μM) efficiently inhibit Tau aggregation and show a low cytotoxicity in cell-based assays [77,78], in C. elegans [35] or in hippocampal slice culture models of tauopathy [79].

### General effects of MB on cell metabolism

The fact that MB-treated WT mice show a somewhat better learning performance compared to untreated WT mice argues for a general increase in cell metabolism or clearance of toxic proteins upon MB treatment. Especially modulation of autophagy and subsequent protein degradation via lysosomes is a promising therapeutic target in neurodegenerative diseases since autophagy is a major pathway for the clearance of long-lived and aggregated proteins [80,81]. Indeed, aggregated forms of Tau are cleared preferentially by autophagy [82]; however, the situation is less well defined for toxic oligomeric forms of Tau where both degradation pathways may contribute [83]. Several studies suggest an effect of MB on different protein degradation pathways. While Congdon and colleagues show an mTOR-dependent induction of macroautophagy mediated by MB [44], Medina et al. (2011) report an increase of chymotrypsin- and trypsin-like activities of the proteasome after MB treatment. In addition, inhibition of the Hsp70 ATPase activity by MB facilitated chaperone-mediated clearance of Tau [73].

The upregulation of different degradation mechanisms by MB was also observed in our experiments, as judged by different marker proteins. While upregulation of beclin is related to increased macroautophagy, higher levels of HSC70 and Lamp2a argue for an induction of CMA, and the rise in PSMD13 points to an increase of proteasome activity. In this respect, MB provides additional beneficial effects which may enhance MB’s neuroprotective properties. In contrast we were not able to confirm a protective effect of MB in terms of oxidative stress as described by others [62]. These discrepancies may be due to the use of another transgenic mouse model, which differs by cognitive phenotype as well as in brain pathology.

### Importance of the dose

We and others [62] observed that high doses of MB (40 mg/kg/day) are detrimental and rather increase pathology. A hormetic dose-response to MB is discussed in the literature, suggesting that MB’s beneficial effect may be limited to a certain range of concentration, whereas concentrations below or beyond the optimal range are ineffective or even lead to detrimental effects. In rats, an optimum concentration of 4 mg/kg improves cognitive function, whereas lower (1 mg/kg) and higher (10 mg/kg) doses were ineffective [84]. In addition, an excessive dose of 50 mg/kg resulted in a decrease of cognitive functions below control levels, indicating a detrimental effect of MB [84]. This hormetic dose-response was attributed to MB’s auto-oxidizing properties. In vivo at low concentrations (<5 μM), reduced MBH_2_ and oxidized MB are in equilibrium and form a reversible reduction-oxidation system, which functions as an electron cycler and scavenger of free radicals. By this mechanism, MB has the potential to donate electrons to the mitochondrial respiratory chain, which finally increases energy production. On the contrary, higher MB concentrations (>5-10 μM) eliminate the equilibrium and electrons are taken away from the respiratory chain. This results in inhibition of cytochrome c oxidation, either directly via reduction of oxygen [85] or indirectly via inhibition of nitric oxide synthase (NO) [86].

Currently attempts are underway to further improve MB in terms of bioavailability and tolerance. A new formulation of Rember™ named LMTX™ provides MB in a stabilized, reduced “leuco-form”, which shows a higher absorbance from the intestine and therefore increases the effective dose of MB in the brain [4,33,34]. Another strategy is the development of MB-loaded hydrophobic nanoparticles suitable for blood-brain barrier permeation, which may provide a steady-state drug release instead of variable drug concentrations after intake of a single daily dose [87].

## Conclusion

In summary, we observed several effects of MB, which may together contribute to the improvement of cognition in treated Tau-transgenic mice. The most crucial parameters in our experiments are (i) the time point of intervention and (ii) the aggregation propensity of Tau. A preventive strategy (initiated before onset of cognitive decline) can be successful, provided that the aggregation propensity is not too strong, which is the case for full-length Tau^ΔK^ but not for TauRD^ΔK^. However, in neither case can the pathology be reversed by MB if therapeutic treatment is started after the onset of cognitive decline.

In other words, MB treatment is no longer effective when the destruction of synaptic functions has already progressed beyond the “point of no return”. The direct effect of MB on Tau aggregation is one of the key events in the defense against Tau-induced functional changes since MB’s potency decreases with increasing β-propensity of Tau. Additionally, beneficial side effects of MB that enhance neuronal cell metabolism reinforce the neuroprotective impact of MB.

Thus, our study supports the use of MB as Tau aggregation inhibitor for treatment of AD and other tauopathies but clearly emphasizes the need for early preventive intervention prior to irreversible synaptic damage.

## Additional files

Additional file 1: Figure S1. Domains of human full-length Tau^ΔK^ and repeat domain TauRD^ΔK^. Diagram of the domains of human full-length Tau^ΔK^ (2N4R, amino acids 1-441) and repeat domain TauRD^ΔK^ (amino acid 244-372). A pro-aggregant variant of Tau^ΔK^ or TauRD^ΔK^ with deletion of lysine 280 (ΔK280) is expressed in inducible Tau transgenic mice. The hexapeptide motifs (PHF6*, PHF6) are located in the repeat domain (R1-R4) and show a high tendency to form β-structure. Adapted from [27]. Additional file 2: Figure S2. Pharmacokinetic analysis of MB after intravenous and oral application. (a) Profiles of TMT-ion concentrations after intravenous (i.v.) application of 30 mg/kg MB in plasma (ng/ml, red circles) and brain tissue (ng/g, blue squares) measured by LC-MS/MS over 24 h. For i.v. application, TMT-ion half-life times of t_½_ = 4.4 h in plasma and t_½_ = 3.0 h in brain are determined. (b) Profiles of TMT-ion concentrations after oral administration of 45 mg/kg MB in plasma (ng/ml, red circles) and brain tissue (ng/g, blue squares) measured by LC-MS/MS over 24 h. For oral administration, TMT-ion half-life times of t_½_ = 30.6 h in brain and t_½_ = 4.7 h in plasma are calculated. Final TMT-ion concentrations at 24 h post application are given in nM for each curve. Data represents mean values ± SEM, n = 3 per time point. MB: methylene blue, TMT-ion: tetramethylionium-ion (= MB without chloride and 3*H_2_O), LC-MS/MS: liquid chromatography – mass spectrometry. Additional file 3: Figure S3. Open field activity of MB-treated mice within intervals. (a) Open field activity within 3 intervals à 5 min. Untreated Tau^ΔK^ mice show a decreased exploration behavior in comparison to WT. Mice treated with MB for (a) 14.5mo and (b) 6mo show a considerably higher activity within interval 2 and 3 as compared to untreated Tau^ΔK^ mice, whereas no difference between MB-treated and untreated mice is observed for 3mo of MB application (c). Bars represent mean values ± SEM. Additional file 4: Figure S4. MB application does not influence anxiety-related parameters. No differences are detected between untreated and MB-treated Tau^ΔK^ mice for anxiety-related parameters such as average distance to wall (a) and total time in center (b) throughout all experimental conditions, suggesting that MB does not exert anxiolytic properties. Bars represent mean values ± SEM. Statistics: one-way analysis of variances with post-hoc Newman-Keuls multiple comparisons test. n.s.: not significant. Additional file 5: Figure S5. MWM long-time probe trial - localization of the target platform. Preventive treatment with MB (14.5mo or 6mo) results in a higher preference of the target quadrant in a long term probe trial (a) and in a more precise localization of the target platform during probe trials as indicated by time in target platform (b). In contrast therapeutic MB application for 3mo does not increase the overall preference of MB-treated Tau^ΔK^ mice for the target quadrant, albeit it seems to improve the ability to localize the platform position to some extent, suggesting a minor effect of MB. Bars represent mean values ± SEM. Statistics: (a) two-tailed one sample t-test against chance level of 25%; *: p < 0.05; **: p < 0.01; ***: p < 0.001; (b) two-way repeated measure analysis of variances with post hoc Fishers LSD multiple comparisons test. T: target quadrant; R: right quadrant; O: opposite quadrant; L: left quadrant; PT: probe trial; LTPT: long-term probe trial. Additional file 6: Figure S6. Histological analysis of conformationally changed and phosphorylated Tau in MB-treated mice. (a-e) MC1 staining (epitope 5-15 + 312-322) indicates a pathological Tau conformation in somatosensory cortex neurons of untreated Tau^ΔK^ mice with missorting of Tau to cell soma and apical dendrites (b1-b3). In contrast MB treatment for 14.5mo (c1-c3), 6mo (d1-d3) and 3mo (e1-e2) reduces MC1 immunoreactivity in Tau^ΔK^ mice. Wild-type (WT) animals are MC1-negative (a1). Scale bar: 50 μm. n = 3 animals per condition. (f-j) Staining of phosphorylated Tau (antibody AT180, dual phosphorylation epitope pThr231 + pSer235) in somatosensory cortex of MB-treated and untreated Tau^ΔK^ mice. Untreated Tau^ΔK^ mice exhibit numerous AT180-positive neurons with massive mislocalization of phosphorylated Tau to cell soma and apical dendrites (g1-g3), whereas MB treatment for 14.5mo (h1-h3), 6mo (i1-i3) and 3mo (j1-j3) diminishes the extent of AT180 phosphorylation. Wild-type (WT) animals are AT180-negative (f1). Scale bar: 50 μm. n = 3 animals per condition. Additional file 7: Figure S7. MB shows no influence on mitochondria in CA3 region of the hippocampus. The intracellular distribution of mitochondria in pyramidal neurons of the hippocampal CA3 region is visualized by fluorescence confocal microscopy. Paraffin sections of wild-type animals (a), untreated Tau^ΔK^ mice (b) and MB-treated Tau^ΔK^ mice (c-e) were fixed and stained with OXPHOS antibody cocktail (DyLight 650, red), which was used as a mitochondrial marker. Nuclei were counterstained with Syto13 (green). No difference of mitochondrial immunoreactivity was observed among WT animals and Tau^ΔK^ mice with or without MB treatment for the time period indicated. Scale bar: 10 μm. Additional file 8: Figure S8. Administration of 20 mg/kg MB for 3mo and 14.5mo does not influence insoluble Tau levels in TauRD^ΔK^ mice. Sarcosyl-extraction of insoluble Tau from cortex tissue of TauRD^ΔK^ mice. MB administration using a daily dose of 20 mg/kg MB for (a) 3mo and (b) 14.5mo does not alter levels of detergent-insoluble Tau in comparison to untreated TauRD^ΔK^ mice as shown by the pan-Tau antibody K9JA. Ab: antibody; mTau: mouse Tau. Additional file 9: Figure S9. MB affects the amount of Tau^4RD^ΔK280 protein in inducible N2a cells. (a) Inhibition of recombinant Tau^3RD^ aggregation by MB monitored in vitro by filter assay and Western blot (adapted from [71]). (b) Examples of confocal micrographs of Tau^4RD^ΔK280 expressing N2a cells after 96 h incubation-/expression time. (c) Western blot of the Tau load in Tau^4RD^ΔK280 expressing N2a cells treated with increasing methylene blue concentrations detected by pan-Tau antibody K9JA (c1) and phosphorylation-depended antibody 12E8 (epitopes pSer262/pSer356) (c2). Expression of Tau^4RD^ΔK280 in N2a cells is induced by addition of doxycycline (1 μg/ml) (Tet-on system) as shown by the pan-Tau antibody K9JA. Monomeric and oligomeric Tau is phosphorylated at KXGS motifs inside the repeat domain as shown by Ab 12E8 (epitope pSer262, pSer356). Incubation of N2a cells with increasing concentrations of MB (25nM, 50nM or 100nM) for 4 days results in a dose-dependent increase of oligomeric Tau species and a subsequent decrease of monomeric Tau^4RD^ΔK280. Monomeric Tau includes the entire Tau repeat domain as well as truncated fragments (F2, F3). (d) Relative quantification of (c1) shows the change in Tau protein densities (monomeric vs. oligomeric Tau) upon treatment of cells with increasing concentrations of MB.

## Abbreviations

Aβ: Amyloid-beta
AD: Alzheimer disease
ALS: Amyotrophic lateral sclerosis
ATP: Adenosine triphosphate
BLI: Bioluminescence imaging
bvFTD: Behavioral variant of frontotemporal dementia
CaMKIIα: Calcium/calmodulin-dependent protein kinase IIα
CMA: Chaperone-mediated autophagy
FDA: Food and Drug Administration
FTD: Frontotemporal dementia
GFAP: Glial fibrillary acidic protein
HSC70: Heat shock cognate protein 70
ΔK280: Tau mutant with deletion of lysine 280
Lamp2a: Lysosome-associated membrane protein 2a
MB: Methylene blue
mo: Months
mTOR: mammalian target of rapamycin (protein kinase)
MWM: Morris water maze
NFT: Neurofibrillary tangle
NMDA: N-methyl D-aspartate
NMR: Nuclear magnetic resonance
PHF: Paired helical filament
PSD95: Postsynaptic density 95
PSMD13: a regulatory subunit of the 26S proteasome
SSCx: Somatosensory cortex
Tau^ΔK^: full-length human Tau (2N4R, 441 residues) with ΔK280 deletion mutation
TauRD^ΔK^: Tau 4-repeat domain (construct K18, residues 244-372) with ΔK280 mutation
tTA: tetracycline transactivator
