# Supplementary figures and images for: Preventive methylene blue treatment preserves cognition in mice expressing full-length pro-aggregant human Tau
Source: Acta Neuropathol Commun. 2015 May 10;3:25. doi: 10.1186/s40478-015-0204-4 (PMC4425867; doi:10.1186/s40478-015-0204-4)

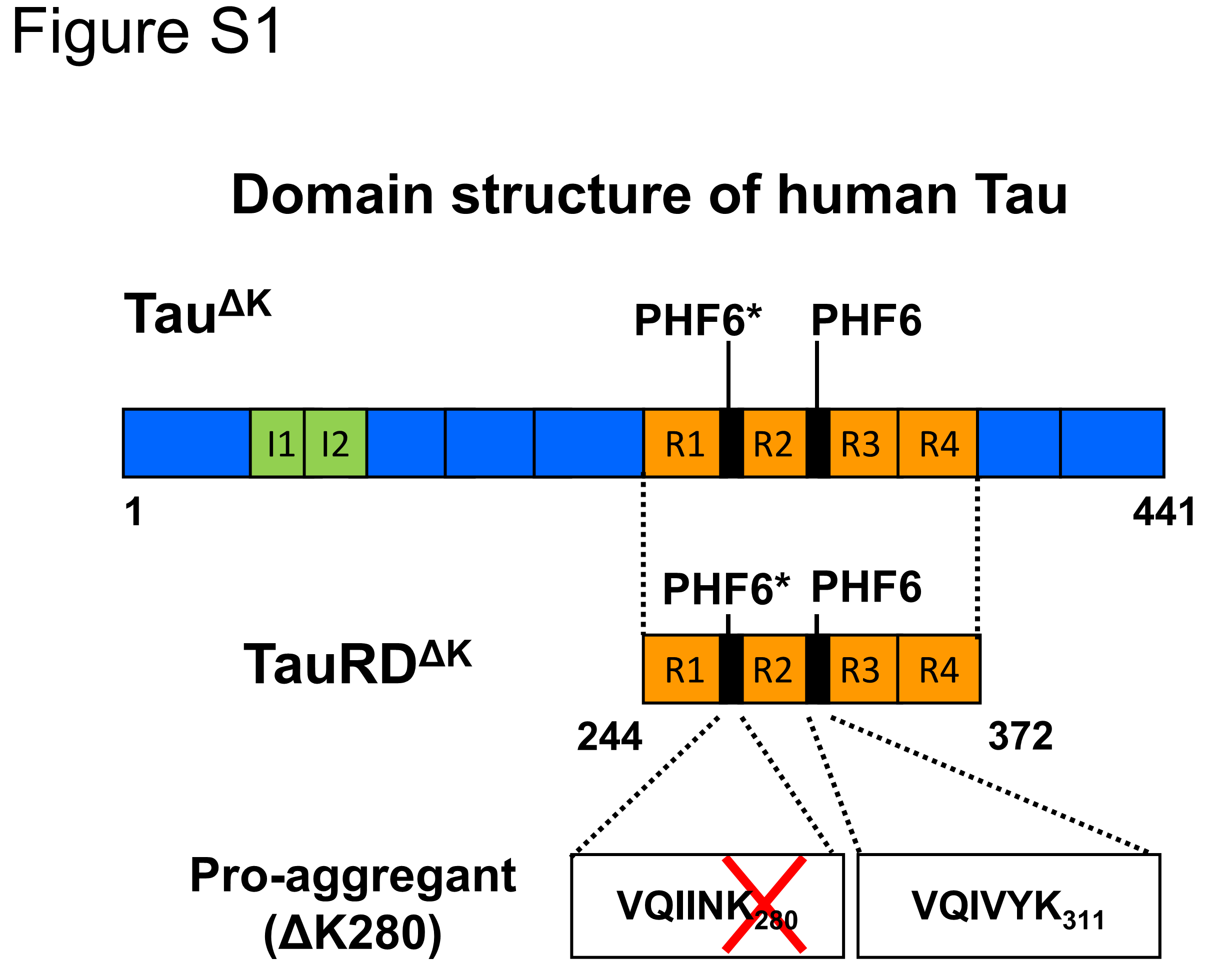

Supplement: Additional file 1: Figure S1. — Domains of human full-length TauΔK and repeat domain TauRDΔK. Diagram of the domains of human full-length TauΔK (2N4R, amino acids 1-441) and repeat domain TauRDΔK (amino acid 244-372). A pro-aggregant variant of TauΔK or TauRDΔK with deletion of lysine 280 (ΔK280) is expressed in inducible Tau transgenic mice. The hexapeptide motifs (PHF6*, PHF6) are located in the repeat domain (R1-R4) and show a high tendency to form β-structure. Adapted from [27]. [file 40478_2015_204_MOESM1_ESM.tif]

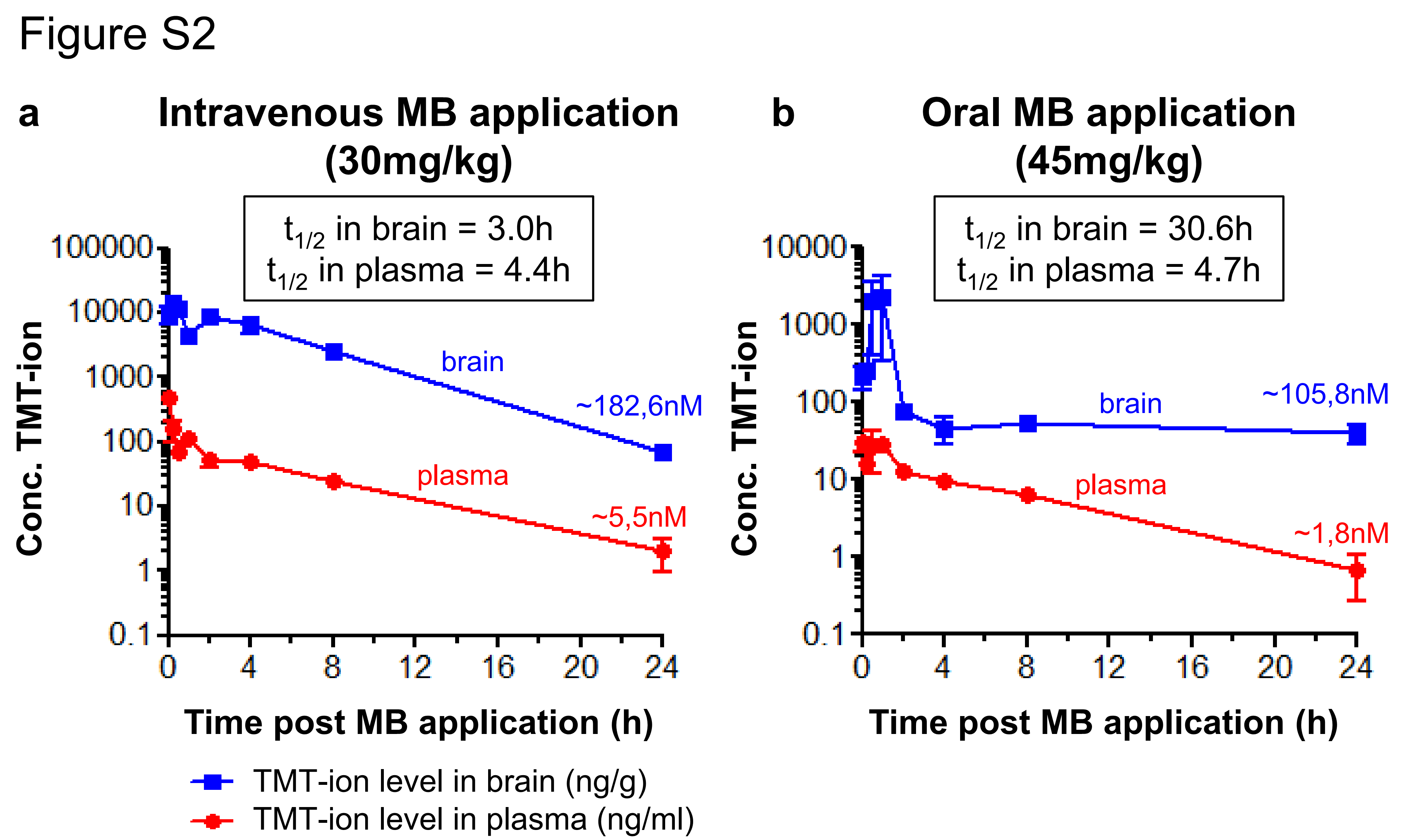

Supplement: Additional file 2: Figure S2. — Pharmacokinetic analysis of MB after intravenous and oral application. (a) Profiles of TMT-ion concentrations after intravenous (i.v.) application of 30 mg/kg MB in plasma (ng/ml, red circles) and brain tissue (ng/g, blue squares) measured by LC-MS/MS over 24 h. For i.v. application, TMT-ion half-life times of t½ = 4.4 h in plasma and t½ = 3.0 h in brain are determined. (b) Profiles of TMT-ion concentrations after oral administration of 45 mg/kg MB in plasma (ng/ml, red circles) and brain tissue (ng/g, blue squares) measured by LC-MS/MS over 24 h. For oral administration, TMT-ion half-life times of t½ = 30.6 h in brain and t½ = 4.7 h in plasma are calculated. Final TMT-ion concentrations at 24 h post application are given in nM for each curve. Data represents mean values ± SEM, n = 3 per time point. MB: methylene blue, TMT-ion: tetramethylionium-ion (= MB without chloride and 3*H2O), LC-MS/MS: liquid chromatography – mass spectrometry. [file 40478_2015_204_MOESM2_ESM.tif]

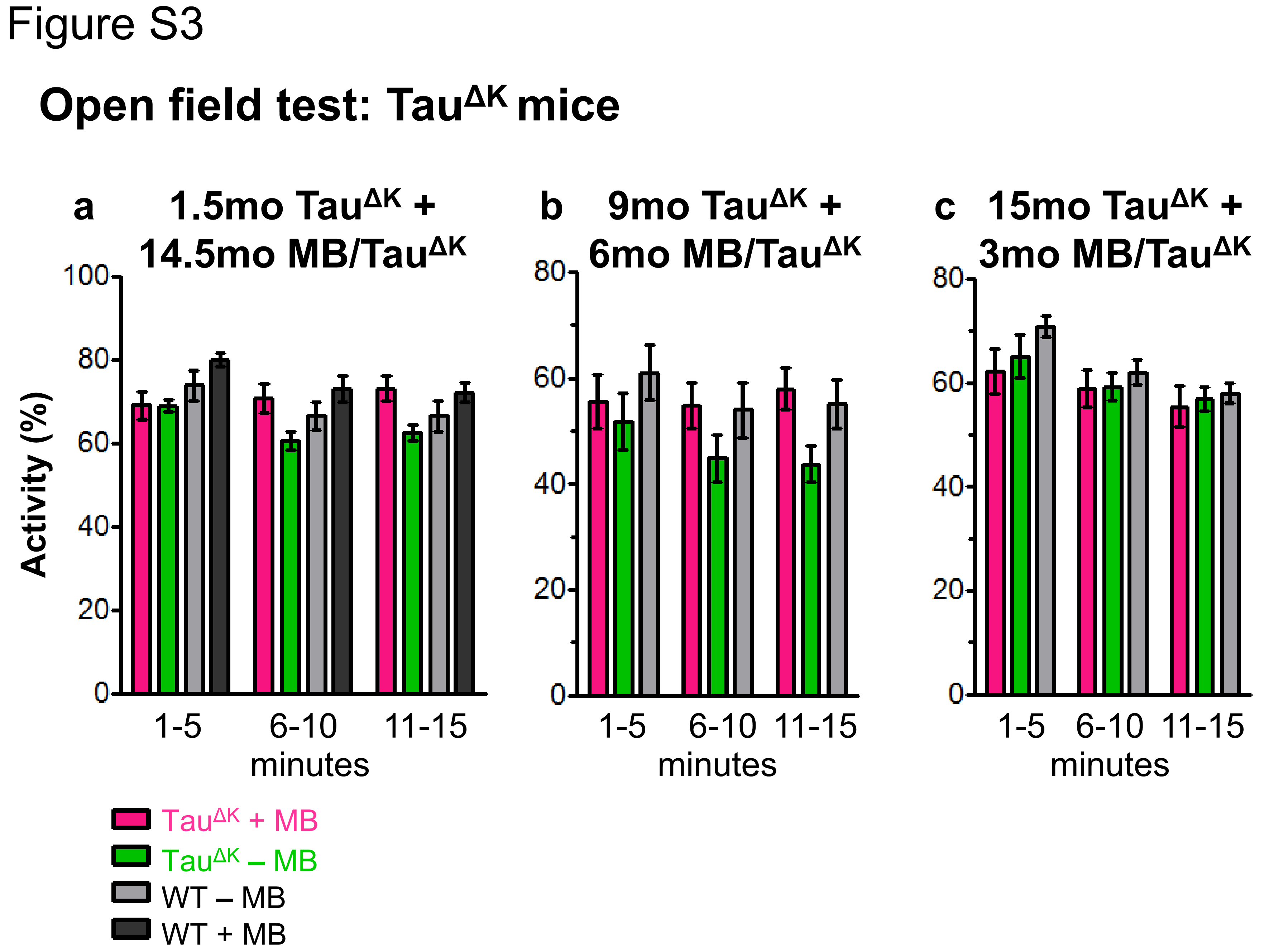

Supplement: Additional file 3: Figure S3. — Open field activity of MB-treated mice within intervals. (a) Open field activity within 3 intervals à 5 min. Untreated TauΔK mice show a decreased exploration behavior in comparison to WT. Mice treated with MB for (a) 14.5mo and (b) 6mo show a considerably higher activity within interval 2 and 3 as compared to untreated TauΔK mice, whereas no difference between MB-treated and untreated mice is observed for 3mo of MB application (c). Bars represent mean values ± SEM. [file 40478_2015_204_MOESM3_ESM.tif]

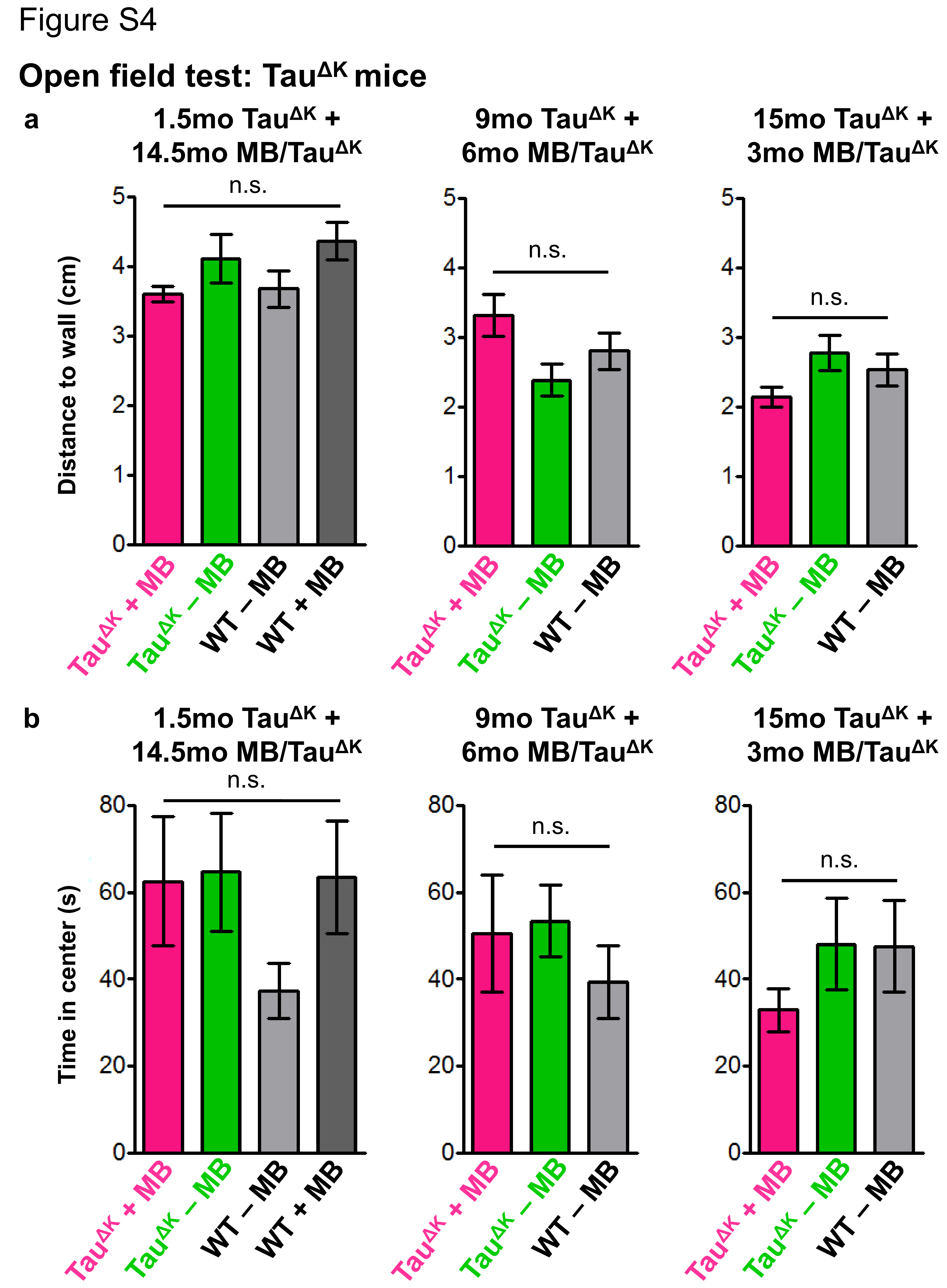

Supplement: Additional file 4: Figure S4. — MB application does not influence anxiety-related parameters. No differences are detected between untreated and MB-treated TauΔK mice for anxiety-related parameters such as average distance to wall (a) and total time in center (b) throughout all experimental conditions, suggesting that MB does not exert anxiolytic properties. Bars represent mean values ± SEM. Statistics: one-way analysis of variances with post-hoc Newman-Keuls multiple comparisons test. n.s.: not significant. [file 40478_2015_204_MOESM4_ESM.tif]

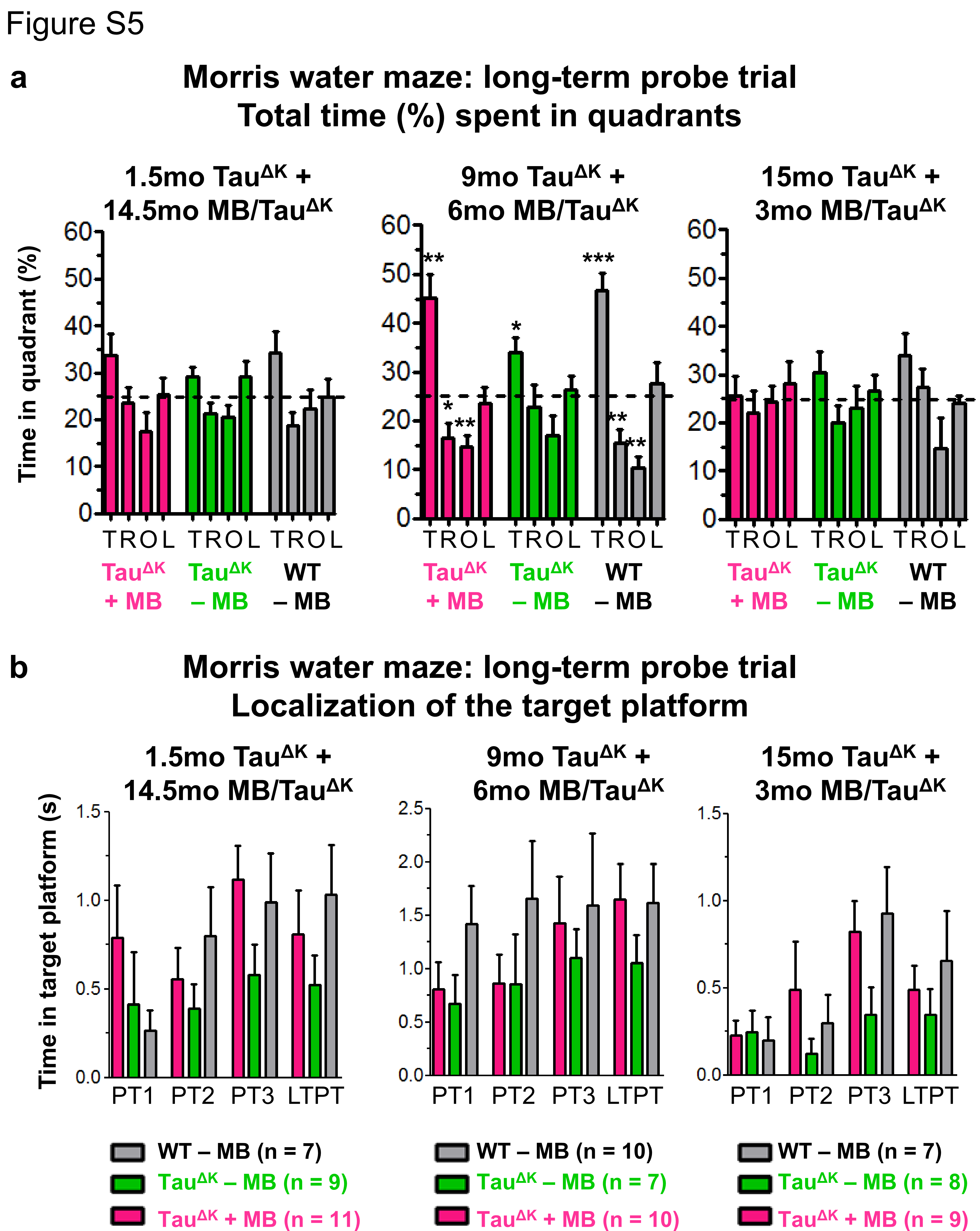

Supplement: Additional file 5: Figure S5. — MWM long-time probe trial - localization of the target platform. Preventive treatment with MB (14.5mo or 6mo) results in a higher preference of the target quadrant in a long term probe trial (a) and in a more precise localization of the target platform during probe trials as indicated by time in target platform (b). In contrast therapeutic MB application for 3mo does not increase the overall preference of MB-treated TauΔK mice for the target quadrant, albeit it seems to improve the ability to localize the platform position to some extent, suggesting a minor effect of MB. Bars represent mean values ± SEM. Statistics: (a) two-tailed one sample t-test against chance level of 25%; *: p < 0.05; **: p < 0.01; ***: p < 0.001; (b) two-way repeated measure analysis of variances with post hoc Fishers LSD multiple comparisons test. T: target quadrant; R: right quadrant; O: opposite quadrant; L: left quadrant; PT: probe trial; LTPT: long-term probe trial. [file 40478_2015_204_MOESM5_ESM.tif]

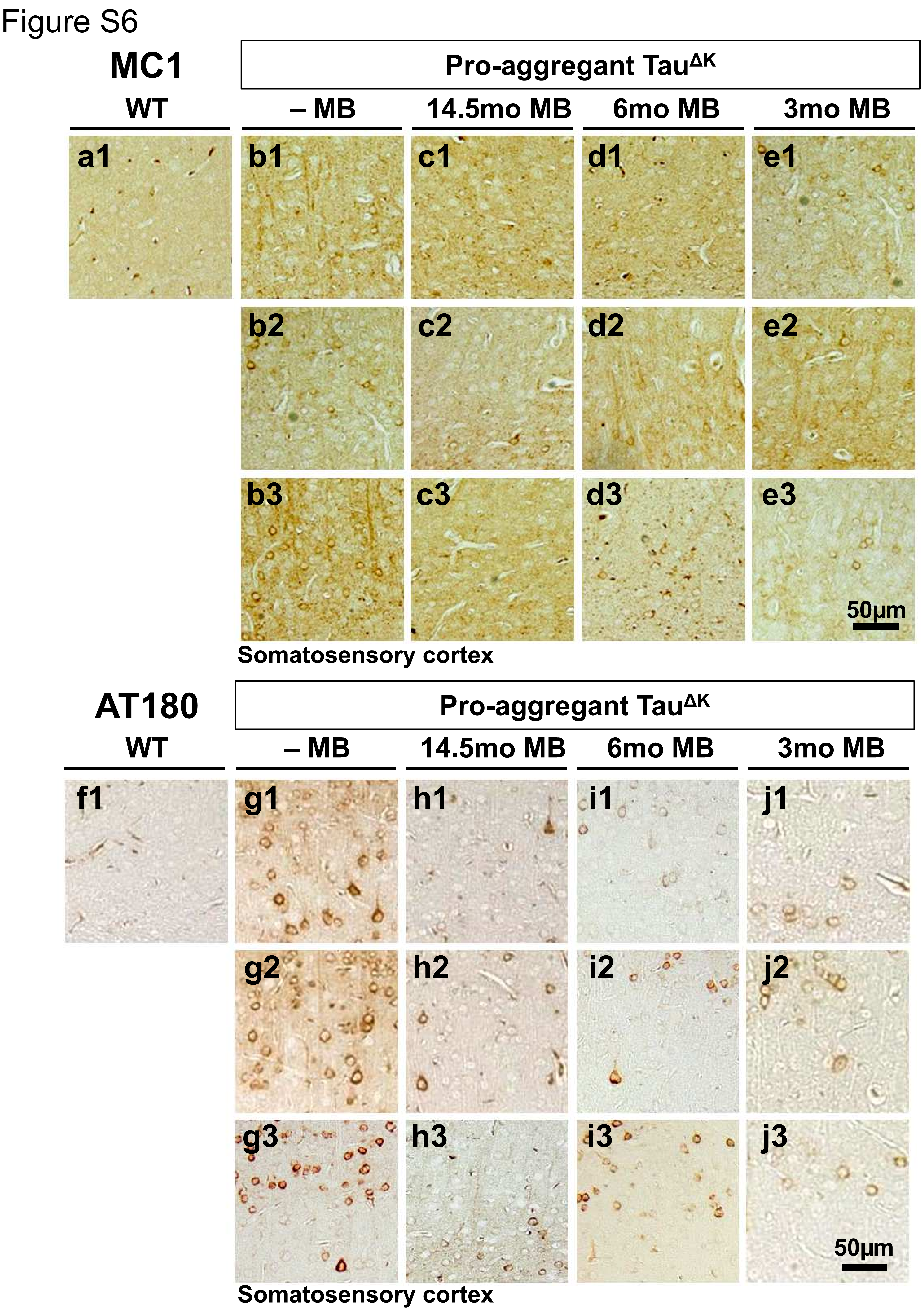

Supplement: Additional file 6: Figure S6. — Histological analysis of conformationally changed and phosphorylated Tau in MB-treated mice. (a-e) MC1 staining (epitope 5-15 + 312-322) indicates a pathological Tau conformation in somatosensory cortex neurons of untreated TauΔK mice with missorting of Tau to cell soma and apical dendrites (b1-b3). In contrast MB treatment for 14.5mo (c1-c3), 6mo (d1-d3) and 3mo (e1-e2) reduces MC1 immunoreactivity in TauΔK mice. Wild-type (WT) animals are MC1-negative (a1). Scale bar: 50 μm. n = 3 animals per condition. (f-j) Staining of phosphorylated Tau (antibody AT180, dual phosphorylation epitope pThr231 + pSer235) in somatosensory cortex of MB-treated and untreated TauΔK mice. Untreated TauΔK mice exhibit numerous AT180-positive neurons with massive mislocalization of phosphorylated Tau to cell soma and apical dendrites (g1-g3), whereas MB treatment for 14.5mo (h1-h3), 6mo (i1-i3) and 3mo (j1-j3) diminishes the extent of AT180 phosphorylation. Wild-type (WT) animals are AT180-negative (f1). Scale bar: 50 μm. n = 3 animals per condition. [file 40478_2015_204_MOESM6_ESM.tif]

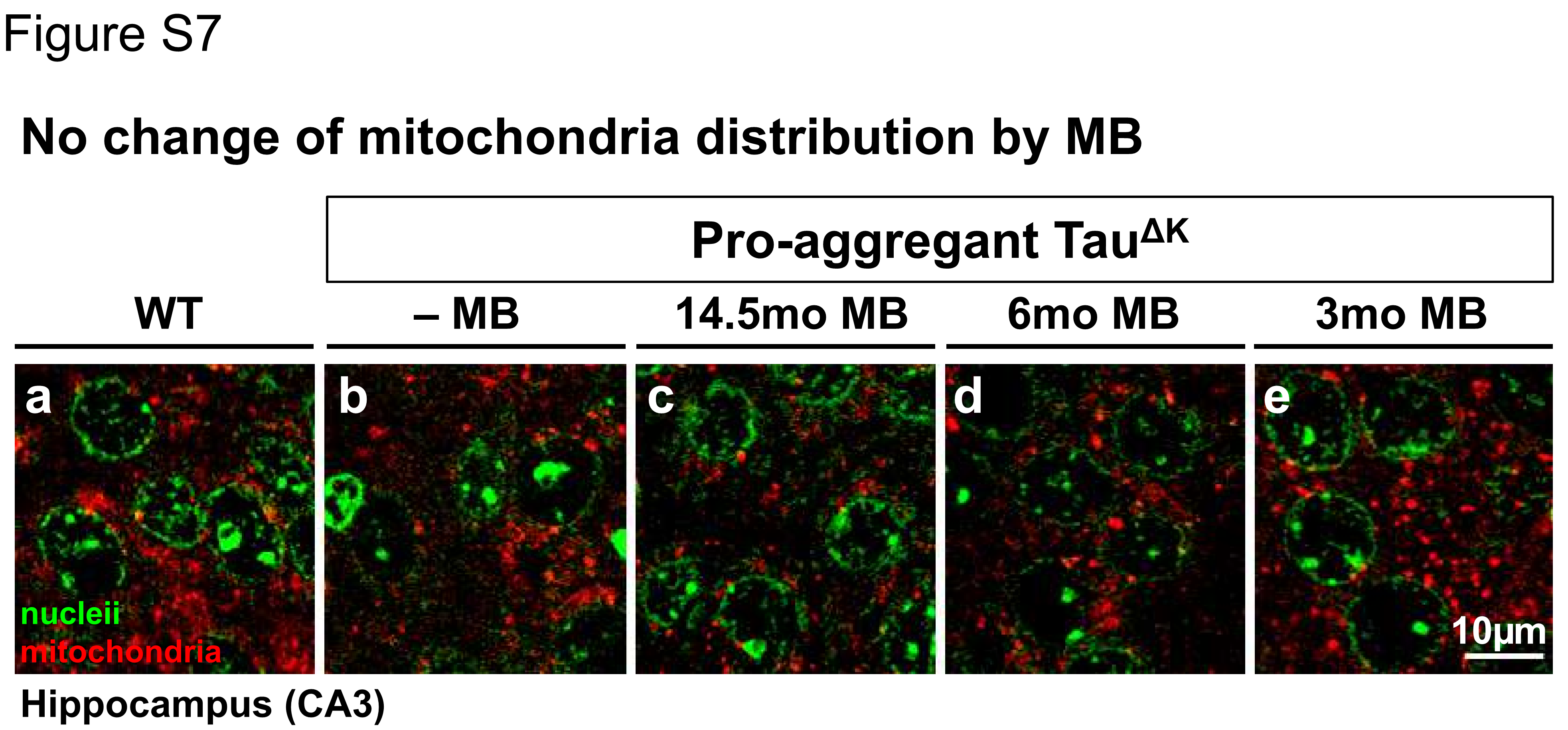

Supplement: Additional file 7: Figure S7. — MB shows no influence on mitochondria in CA3 region of the hippocampus. The intracellular distribution of mitochondria in pyramidal neurons of the hippocampal CA3 region is visualized by fluorescence confocal microscopy. Paraffin sections of wild-type animals (a), untreated TauΔK mice (b) and MB-treated TauΔK mice (c-e) were fixed and stained with OXPHOS antibody cocktail (DyLight 650, red), which was used as a mitochondrial marker. Nuclei were counterstained with Syto13 (green). No difference of mitochondrial immunoreactivity was observed among WT animals and TauΔK mice with or without MB treatment for the time period indicated. Scale bar: 10 μm. [file 40478_2015_204_MOESM7_ESM.tif]

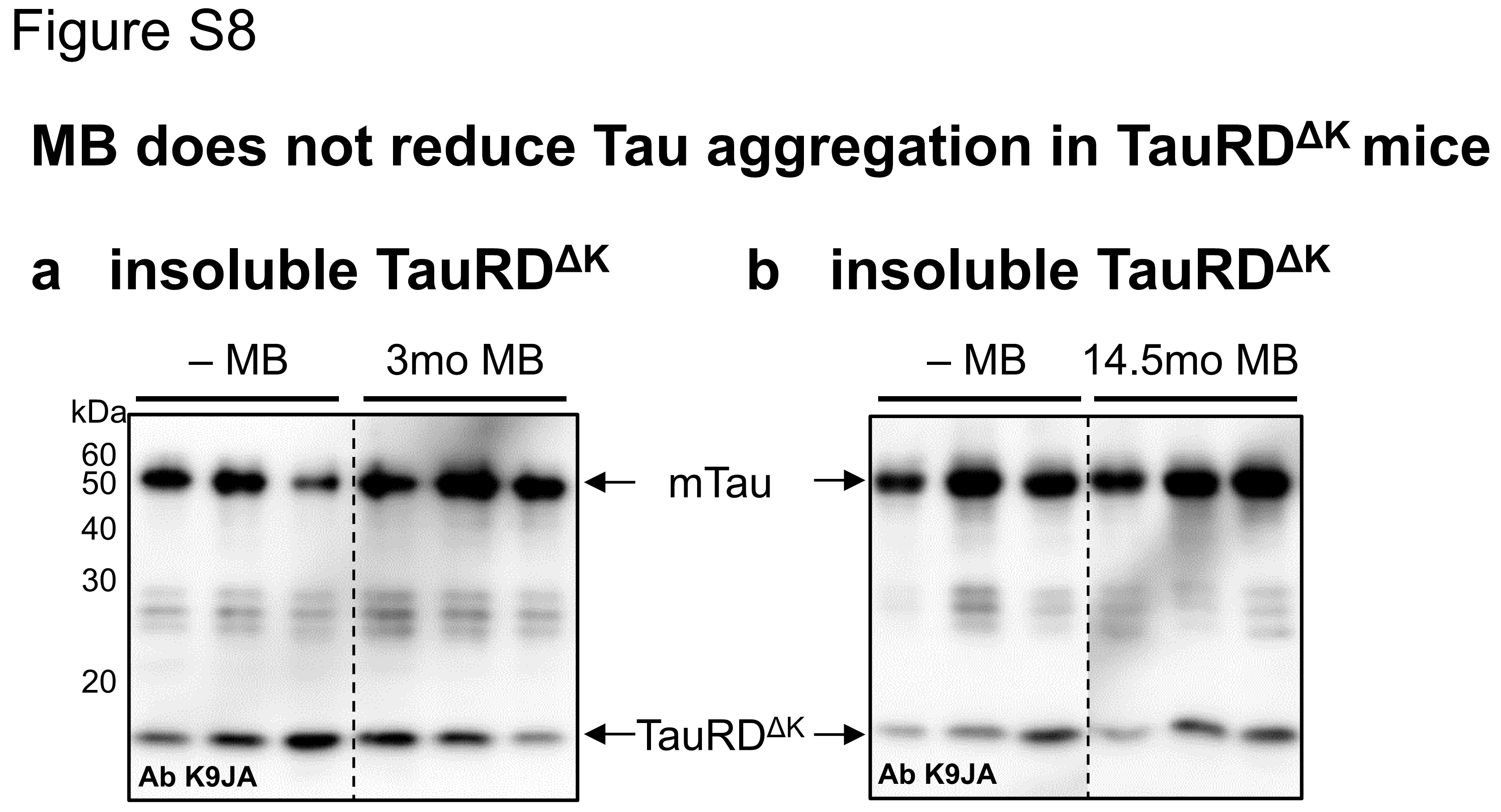

Supplement: Additional file 8: Figure S8. — Administration of 20 mg/kg MB for 3mo and 14.5mo does not influence insoluble Tau levels in TauRDΔK mice. Sarcosyl-extraction of insoluble Tau from cortex tissue of TauRDΔK mice. MB administration using a daily dose of 20 mg/kg MB for (a) 3mo and (b) 14.5mo does not alter levels of detergent-insoluble Tau in comparison to untreated TauRDΔK mice as shown by the pan-Tau antibody K9JA. Ab: antibody; mTau: mouse Tau. [file 40478_2015_204_MOESM8_ESM.tif]

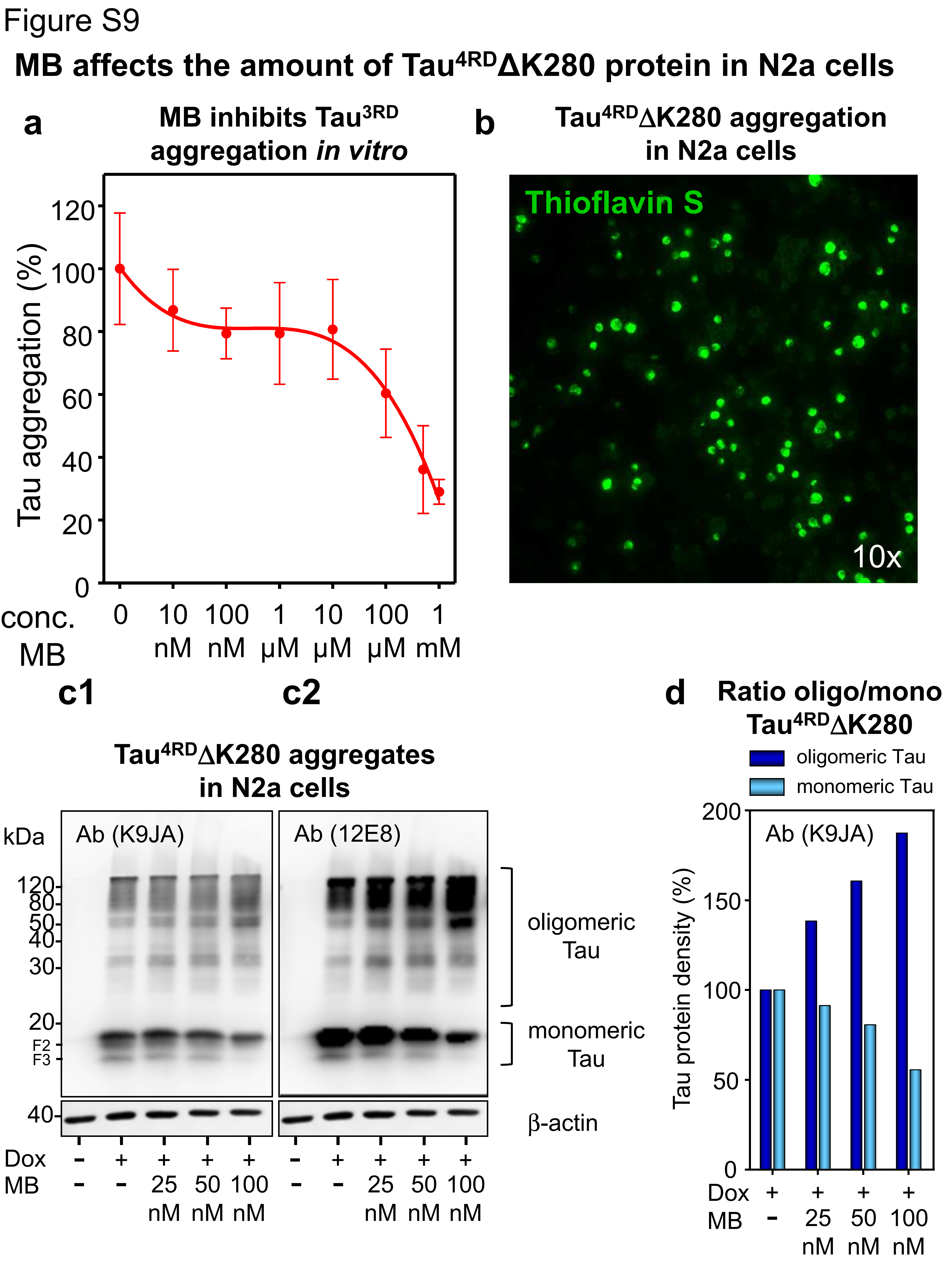

Supplement: Additional file 9: Figure S9. — MB affects the amount of Tau4RDΔK280 protein in inducible N2a cells. (a) Inhibition of recombinant Tau3RD aggregation by MB monitored in vitro by filter assay and Western blot (adapted from [71]). (b) Examples of confocal micrographs of Tau4RDΔK280 expressing N2a cells after 96 h incubation-/expression time. (c) Western blot of the Tau load in Tau4RDΔK280 expressing N2a cells treated with increasing methylene blue concentrations detected by pan-Tau antibody K9JA (c1) and phosphorylation-depended antibody 12E8 (epitopes pSer262/pSer356) (c2). Expression of Tau4RDΔK280 in N2a cells is induced by addition of doxycycline (1 μg/ml) (Tet-on system) as shown by the pan-Tau antibody K9JA. Monomeric and oligomeric Tau is phosphorylated at KXGS motifs inside the repeat domain as shown by Ab 12E8 (epitope pSer262, pSer356). Incubation of N2a cells with increasing concentrations of MB (25nM, 50nM or 100nM) for 4 days results in a dose-dependent increase of oligomeric Tau species and a subsequent decrease of monomeric Tau4RDΔK280. Monomeric Tau includes the entire Tau repeat domain as well as truncated fragments (F2, F3). (d) Relative quantification of (c1) shows the change in Tau protein densities (monomeric vs. oligomeric Tau) upon treatment of cells with increasing concentrations of MB. [file 40478_2015_204_MOESM9_ESM.tif]
